# Crotonylation driving *Streptococcu*s *pneumoniae* adaption and virulence

**DOI:** 10.1016/j.jare.2025.06.045

**Published:** 2025-06-19

**Authors:** Nan Li, Jianpeng Zhuang, Jiayi Wu, Zhuoti Xue, Jiayi Xu, Zuye Fang, Yundan Zheng, Yun Liu, Yunpeng Yang, Xinyu Ye, Qing-Yu He, Xuesong Sun

**Affiliations:** MOE Key Laboratory of Tumor Molecular Biology and State Key Laboratory of Bioactive Molecules and Druggability Assessment, Institute of Life and Health Engineering, College of Life Science and Technology, Jinan University, Guangzhou, China

**Keywords:** Crotonylation, *Streptococcus pneumoniae*, Pneumolysin, Bacterial virulence

## Abstract

**Introduction:**

To cope with the host’s stressful environment and immune clearance, some drug-resistant bacteria have reduced their virulence. Protein post-translational modifications (PTMs) are known to regulate numerous critical biological processes; however, their specific role in modulating bacterial virulence remains poorly understood.

**Objective:**

This study seeks to elucidate the mechanistic role of lysine crotonylation (Kcr) in modulating *Streptococcus pneumoniae* virulence and adaptive survival.

**Methods:**

This study employed quantitative differential proteomics combined with animal models to delineate the critical role of Kcr in the adaptive survival of clinically multidrug-resistant bacterial strains. A crotonyltransferase was identified via knockout/overexpression screening, and its regulatory role was characterized using multi-omics characterization methods, including biochemical and molecular biology assays. Antibody-based enrichment coupled with proteomic techniques was utilized to map the enzyme-substrate network of the crotonyltransferase while comprehensive biochemical analyses-including isothermal titration calorimetry (ITC), hemolysis assays, immunoblotting, circular dichroism (CD) spectroscopy and molecular docking, deciphered its mechanism in modulating the virulence effector pneumolysin (PLY).

**Results:**

Our results reveal that mice infected with clinically multidrug-resistant *S.pn* strains exhibit enhanced invasiveness but attenuated virulence. Quantitative proteomics reveal that Kcr serves as a critical regulator of bacterial virulence and host adaptation. SPD_0839 functions as a crotonyltransferase in *S.pn*, with Phe93 as its critical catalytic residue. SPD_0839 upregulates 205 Kcr sites on 153 substrates, enhancing the Kcr levels of key enzymes involved in energy metabolism, thereby regulating ATP production. Notably, SPD_0839 modulates the function of the key virulence factor PLY by catalyzing Kcr at Lys171 and Lys442, increases its pore-forming activity and ultimately enhances bacterial hemolysis activity and virulence.

**Conclusion:**

Our findings shed light on the molecular mechanisms underlying PLY hemolytic activity regulated by Kcr, revealing its importance in bacterial survival and virulence. Overall, this research provides valuable insights into the important biological roles of Kcr in *S.pn* and offers potential theoretical foundations for developing new antibacterial drugs.

## Highlights

• Crotonylation regulates the survival and adaptation of *Streptococcus pneumoniae* (*S.pn*) in host.
• SPD_0839 functions as a novel crotonyltransferase in *S.pn*.
• Crotonylation proteomics reveals key endogenous substrate proteins of SPD_0839 involved in metabolism and virulence.
• SPD_0839 alters the bacterial hemolysis activity and virulence via crotonylation of pneumolysin.
• SPD_0839 may be potential novel therapeutic targets for combating *S.pn* infections.

## Graphical abstract

The diagram illustrates the role of SPD_0839 as a crotonyltransferase mediating crotonylation on key endogenous substrates, influencing energy metabolism, hemolysis activity, and bacterial virulence, which collectively contribute to bacterial survival and adaptation in the host environment.

**Figure:**
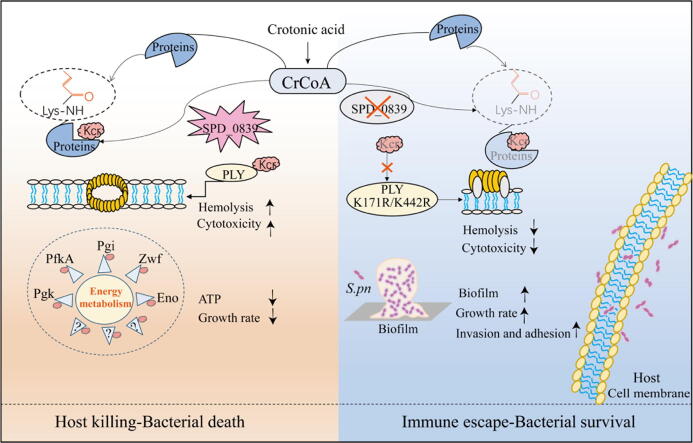

## Introduction

*Streptococcus pneumoniae* (*S.pn*), a highly adaptable Gram-positive pathogen, causes a wide range of serious illnesses, including otitis media, bronchitis, meningitis and sepsis, and is a major contributor to pneumonia-related mortality [[1], [2], [3]]. Adhesion and invasion are key steps in the infection process, facilitated by various virulence factors that enable *S.pn* to colonize the nasopharynx and evade host immune defences [4]. One important virulence factor is pneumolysin (PLY), which binds cholesterol in host cell membranes, causing cell lysis and tissue damage [5,6]. Although antibiotics and vaccines are the primary strategies for managing *S.pn* infections, the pathogen's genetic diversity and adaptability complicate efforts to address the interplay between resistance and virulence [7,8]. The rapid rise in drug-resistant strains highlights the urgent need for novel therapeutic strategies to combat *S.pn* infections.

Post-translational modifications (PTMs) play a critical role in regulating protein function, influencing numerous biological processes. To date, a wide variety of PTMs, including phosphorylation, acetylation, and succinylation, have been identified in hundreds of bacterial species, with evidence demonstrating their impact on bacterial physiology and pathogenesis [[9], [10], [11], [12], [13]]. This has led to the hypothesis that PTMs are crucial for regulating bacterial resistance and virulence during adaptation to host environments. Therefore, exploring the PTMs regulatory mechanism of bacterial virulence in host adaptive survival will help solve the current serious public health problem of bacterial infections.

Lysine crotonylation (Kcr), a recently discovered PTM, was first identified on lysine residues of human histones [14]. Kcr has been shown to regulate diverse processes by altering histone structure and function, including tumor immunity, spermatogenesis, endoderm differentiation, HIV latency, and cancer progression [[15], [16], [17], [18], [19]]. Over the past decade, advances in high-resolution liquid chromatography-tandem mass spectrometry (LC-MS/MS) have revealed Kcr modifications on non-histone proteins involved in nearly all biological processes. Kcr is a reversible modification mediated by crotonyltransferases (“writers”) and decrotonylases (“erasers”). In mammals, Kcr “writers” have been identified within three major histone acetyltransferase (HAT) families: p300/CREB-binding protein (p300/CBP), MOZ-YBF2/SAS3-SAS2-TIP60 (MYST), and Gcn5-related N-acetyltransferase (GNAT). Additionally, MYST family proteins, such as human MOFs and yeast Esa1, exhibit significant crotonyltransferase activity on histones H3 and H4 [[20], [21], [22]]. Eukaryotic “erasers” include silencing regulatory proteins like Sirt1 and Sirt3, as well as Class I histone deacetylases (HDACs) such as HDAC1, HDAC3, and HDAC8, which remove the crotonyl group from histones [23,24]. Similarly, the decrotonylase FoSir5 in Fusarium oxysporum regulates germination by removing Kcr from histone H3K18.

Current studies have revealed crotonylome profiles in four bacterial species including *Streptococcus agalactiae* (675 Kcr sites and 241 Kcr proteins), *Brucella* (5709 Kcr sites and 1290 Kcr proteins), *Streptomyces roseosporus* (3944 Kcr sites and 1389 Kcr proteins) *and Escherichia coli* (5675 Kcr sites and 1324 Kcr proteins). Additionally, a total of two “writer” enzymes BspF and *kct*1 have been identified in *Brucella* and *Streptomyces roseosporus* separately, while two “eraser” enzymes CobB and HDAC were characterized in *Streptomyces* species [[25], [26], [27], [28], [29], [30]]. Identifying Kcr “writers” and “erasers” in prokaryotes remains challenging due to the lack of sequence homology with eukaryotic enzymes and significant sequence variability among bacterial species. Recently, our group identified YjgM as a crotonyltransferase in *Escherichia coli*, which regulates polymyxin resistance [29]. Additionally, we investigated the lysine acetylome in *S.pn* and *E. coli* and its role in regulating virulence and drug resistance [12,31]. Given the unique structural properties of the crotonyl group, Kcr likely has distinct functional roles compared to lysine acetylation (Kac).

In this study, we aim to comprehensively investigate the role of Kcr in regulating *S.pn* virulence and adaptive survival. Through functional screening, we first identified SPD_0839 as a novel crotonyltransferase in *S.pn* and elucidated its key endogenous substrates. Our findings shed light on the molecular mechanisms underlying PLY hemolytic activity regulated by Kcr, revealing its importance in bacterial survival and virulence. Overall, this research provides valuable insights into the important biological roles of Kcr in *S.pn* and offers potential theoretical foundations for developing new antibacterial drugs.

## Materials and methods

### Bacterial strains and culture

This study utilized serotype 2 *Streptococcus pneumoniae* (*S.pn*) strain D39. Gene overexpressing strains were constructed by linking target genes to shuttle plasmid 169, followed by transformation into D39 via natural transformation. Gene-deficient strains were generated using homologous recombination, where the target gene was replaced with an erythromycin resistance gene. *S.pn* strains and their derivatives were cultured in THYE broth (Todd-Hewitt broth supplemented with 0.5 % yeast extract) or chemically defined C + Y medium (10 mM Casamino acids, 0.5 % Yeast extract, 0.48 ‰ Bovine serum albumin, 11 mM Glucose, 24 mM Sodium acetate, 50 mM K_2_HPO_4_, 2.5 mM MgCl_2_·6H_2_O, 0.02 mM CaCl_2_, 0.3 mM L-Cysteine, 0.03 mM L-Tryptophan, 0.1 μM MnSO_4_.4H_2_O, 0.002 μM FeSO_4_.7H_2_O, 0.002 μM ZnSO_4_.7H_2_O, 0.002 μM CuSO_4_.5H_2_O, 0.001 μM Biotin, 0.002 mM Nicotinic acid, 0.001 mM Pyridoxine HCl, 0.6 μM Thiamine HCl, 0.27 μM Riboflavin, 0.001 mM Calcium pantothenate, pH 8.0) at 37 °C in an atmosphere containing 5 % CO_2_. For recombinant protein expression, *Escherichia coli* BL21 (λDE3) was employed. A comprehensive list of bacterial strains, plasmids, and primers used in this study can be found in Tables S2 and S3.

### Mouse infection experiments

BALB/c female mice (6 weeks old) were procured from the Experimental Animal Department of Guangzhou Southern Medical University. All experiments adhered to institutional guidelines and were approved by the Animal Experiment Ethics Committee of Jinan University. For the pneumonia infection model, mice were anesthetized with a mixture of 2,2,2-tribromoethanol and 2-methyl-2-butanol. Cultured *S.pn* (OD_600_ = 0.6) strains were intranasally administered in 20 μL PBS droplets, with PBS serving as a negative control. The mice were monitored daily for 14 days and scored for clinical signs. Mice displaying severe symptoms were euthanized by cervical dislocation, and the lung bacterial load was assessed. To measure bacterial load, lungs were homogenized using a sterile grinding rod, and the homogenate was serially diluted with PBS. The dilutions were plated onto Columbia blood agar plates and incubated at 37 °C with 5 % CO_2_. After 12 h, bacterial colonies were counted. Three days post-infection, mice were euthanized to assess lung inflammation.

### Hemolysis assays

In hemolysis assay, bacterial cultures (OD_600_ ∼0.8) were harvested. Subsequently, 100 μL of bacterial supernatant was gently mixed with 100 μL of 20 % (v/v) sheep blood and incubated statically at 37 °C for 1 h. 100 μL of PBS was served as the negative control, while 100 μL of 0.1 % (v/v) Triton X-100 was used as the positive control. After incubation, the mixtures were centrifuged and the supernatant was transferred to a 96-well plate. The absorbance at 543 nm was measured using a microplate reader, and images were captured with a scanner. All experiments were performed in triplicate. In vitro hemolysis assay, 1 μg of purified PLY or its mutant variants was mixed with 100 μL of 20 % (v/v) sheep blood in a 96-well plate and incubated at 37 °C for 15 min. The mixtures were then centrifuged at 2000×g for 5 min at room temperature and the supernatants were transferred to a new 96-well plate, hemolytic activity was determined as described above.

### Construction of D39 strains overexpressing and deficient in specific genes

To construct overexpressing strains, sequences of GNAT family genes in D39 were retrieved from the UniProt database. Primers were designed to amplify the target genes, which were then cloned into the multiple cloning site (MCS) of plasmid 169 using the restriction enzymes *BamH* I and *Sal* I. The recombinant plasmids were transformed into *E. coli* DH5α for amplification, and positive clones were selected on LB plates containing 20 μg/mL chloramphenicol. Verified plasmids were subsequently introduced into competent *S.pn* D39 cells, and monoclonal colonies were selected on Columbia sheep blood agar plates supplemented with 4 μg/mL chloramphenicol.

Gene-deficient strains were generated using the long flanking homology-polymerase chain reaction (LFH-PCR) technique. To achieve this, upstream (500–800 bp) and downstream (500–800 bp) fragments flanking the target gene were amplified and designed to include homologous arms for the erythromycin resistance gene. These fragments, along with the erythromycin resistance cassette, were joined using a two-step PCR reaction. The resulting long DNA fragments were sequenced and transformed into *S.pn* D39. Positive clones were selected on blood plates containing 0.25 μg/mL erythromycin and verified through PCR and sequencing. A detailed list of primers used for strain construction is provided in Supplementary Table 3.

### Expression and purification of recombinant proteins

Recombinant proteins were expressed and purified following established protocols. Briefly, genes were cloned into either the pET-28a or PGEX-4T-1 vectors and transformed into *E. coli* BL21 (λDE3). Transformants were cultured in LB medium supplemented with 50 μg/mL kanamycin (for pET-28a) or 100 μg/mL ampicillin (for PGEX-4T-1). Cultures were incubated at 37 °C in shaking flasks until the optical density at 600 nm (OD_600_) reached 0.6, at which point 0.1 mM isopropyl β-D-1-thiogalactopyranoside (IPTG) was added to induce protein expression. After induction, the cultures were incubated at 20 °C for 20 h. Cells were harvested by centrifugation and lysed using a high-pressure crusher. Soluble proteins containing His-tags or GST-tags were purified from the supernatant using nickel or GST affinity chromatography columns, respectively. Concentrated proteins were obtained using an Amicon Ultra Centrifugal Filter (Millipore). The purity of the recombinant proteins was assessed using SDS-PAGE stained with Coomassie Blue dye.

### Molecular docking analysis

The structure of SPD_0839 was predicted using AlphaFold and retrieved from the UniProt database. Structures for CrCoA and AcCoA were obtained from the PubChem database. Before docking, polar hydrogens were added to the proteins and ligands using AutoDock Tools 1.5.7 (ADT), and files were saved in PDBQT format. Molecular docking was performed with AutoDock Vina using the following parameters: Grid center: X  = -0.044, Y = −0.429, Z = 0.216, Grid box size: X  = 38.25, Y = 36.75, Z = 37.5. The docking process combined local energy search with the Lamarckian genetic algorithm. A semi-empirical scoring function was used to determine ligand binding conformation and position. The configuration with the lowest binding energy was selected as the final docking mode. Docking results were visualized and analyzed using PyMol 2.7.

### Biofilm assay

Biofilm formation was assessed using a previously described protocol. Briefly, bacterial cells were grown to an OD_600_ of 0.6 and inoculated into 24-well (flat-bottom) polystyrene plates at a 1:20 dilution in fresh C + Y medium. Plates were incubated statically at 37 °C in 5 % CO_2_ for 24 h. After incubation, the medium was aspirated, and the wells were washed three times with PBS to remove non-adherent cells. The plates were air-dried at room temperature for 15 min, and biofilms were stained with 1 % crystal violet for 15 min. Excess stain was removed by washing with PBS, and the plates were air-dried. For quantification, the stained biofilm was solubilized with 200 μL of 95 % ethanol per well. Absorbance was measured at 540 nm using a Microplate Spectrophotometer to determine biofilm density.

### Adherence and invasion assays

To evaluate the adhesion and colonization capabilities of *S.pn* D39 and its mutant strains, human A549 lung epithelial cells were used as a model. Adherence and invasion assays were conducted according to established protocols: A total of 2 × 10^5^ A549 cells were seeded in each well of 24-well culture plates and cultured in RPMI 1640 medium supplemented with 10 % fetal bovine serum (FBS) for 24 h to form a monolayer. Approximately 1 × 10^7^ colony-forming units (CFU) of *S.pn* D39 or mutant strains were added to the wells (OD_600_ = 0.6) at a multiplicity of infection (MOI) of 50. Plates were incubated at 37 °C with 5 % CO_2_ for 1 h for adherence assays and 2 h for invasion assays. For adherence assays, cells were incubated with 100 μg/mL gentamicin and 20 μg/mL ampicillin for an additional 2 h to kill non-adherent bacteria. Cells were washed with PBS, then lysed with a solution containing 0.25 % trypsin and 0.025 % Triton X-100. Lysates were serially diluted (10-fold) and plated on Columbia blood agar plates. Colony counts were used to quantify adherent and invasive bacteria.

### Quantification of ATP production

To measure ATP production, *S.pn* D39 was cultured in 10 mL of C + Y medium to an OD_600_ of 0.8. ATP levels were quantified using an improved ATP assay kit (Beyotime, China) following the manufacturer’s instructions. Luminescence was detected using a GloMax luminometer (Promega, USA).

### Intracellular assay of PfkA activity

The activity of Phosphofructokinase A (PfkA) was analyzed using the Phosphofructokinase (PFK) Activity Assay Kit according to the manufacturer’s guidelines. *S.pn* D39 cultures were grown to an OD_600_ of 0.8, and 10 mL of culture was harvested for analysis.

### GST pull-down assay

To identify interacting proteins, GST pull-down assays were performed: Glutathione-Sepharose beads were incubated with either GST or SPD_0839-GST protein for 2 h at 4 °C with rotation. Then beads were incubated overnight at 4 °C with *S.pn* D39 lysates in a 1:5 ratio (beads to lysate). After centrifugation and washing, bound proteins were eluted. Eluted proteins were separated by SDS-PAGE and identified using mass spectrometry.

### Protein sample preparation

To analyze the *S.pn* D39 proteome, including overexpressing strains of SPD_0839 and controls with empty plasmid p169, bacterial samples were processed as follows: bacteria were washed with PBS and lysed using a buffer containing 8 M urea, 1 % Triton X-100, 10 mM dithiothreitol (DTT), 1 % protease inhibitor, 3 mM TSA, 50 mM NAM, and 2 mM EDTA. Samples were then ultrasonicated on ice and lysates were centrifuged at 12,000 g for 30 min at 4 °C, and then 20 % (v/v) trichloroacetic acid was added to the supernatant. Samples were incubated at 4 °C for 2 h or overnight to precipitate proteins. Precipitated proteins were washed three times with pre-cooled acetone and dissolved in 8 M Urea. Proteins were reduced with 5 mM DTT at 56 °C for 30 min and alkylated with 11 mM iodoacetamide at room temperature in the dark for 15 min. Proteins were washed five times with 100 mM TEAB using ultrafiltration tubes to remove urea. Trypsin was added at a 1:50 trypsin-to-protein mass ratio, and digestion was performed overnight at 37 °C. Tryptic peptides were desalted using C18 Zip Tips and vacuum-dried for further analysis.

### Pan-antibody enrichment technique

To enrich crotonylated peptides, 2 mg of tryptic peptides were prepared in NETN buffer (100 mM NaCl, 1 mM EDTA, 50 mM Tris-HCl, 0.5 % Nonidet P-40, pH 8.0). The peptides were incubated with pre-washed antibody-conjugated beads (PTM-503 for Kcr, PTM Bio) at 4 °C overnight with gentle shaking. After incubation, the beads were washed four times using solution buffer II to remove non-specific peptides. Peptide elution was performed in a stepwise manner using solution buffer III. The eluted fractions were pooled and subjected to vacuum centrifugation for drying. Desalting was performed using C18 Zip Tips before peptides were analyzed for identification and quantification by LC-MS/MS.

### HPLC-MS/MS analysis

The tryptic peptides were dissolved in a solution containing 0.1 % formic acid, followed by the addition of the iRT-Standard (Biognosys, MA, USA). Both data-dependent acquisition (DDA) and data-independent acquisition (DIA) proteomics analysis were conducted on an Orbitrap Fusion Lumos mass spectrometer (Thermo Fisher Scientific) according to established protocols. For data-dependent acquisition (DDA), mass spectra were acquired in top-speed mode with the following parameters: MS1 scans were performed over a mass range of 350–1500 *m*/*z* at a resolution of 120,000, using an automatic gain control (AGC) target of 4 × 10^5^ and a maximum injection time of 50 ms. MS2 scans were acquired at a resolution of 15,000 with a 1.6 *m*/*z* isolation window, employing higher-energy collisional dissociation (HCD) at 32 % normalized collision energy. The AGC target for MS2 was set to 5 × 10^4^ with a maximum injection time of 30 ms, using a 3-second cycle time and 30-second dynamic exclusion window. For data-independent acquisition (DIA), MS1 scans were collected from 350-1250 *m*/*z* at 60,000 resolution with an AGC target of 4 × 10^5^ and 50 ms maximum injection time, followed by 40 variable-width DIA scans adjusted according to precursor density. MS2 scans were acquired at 30,000 resolution with an AGC target of 5 × 10^5^, 56 ms maximum injection time, and HCD collision energy set to 32 %. Data Analysis: Raw LC-MS/MS data were analyzed against the UniProtKB *S.pn* D39 proteome sequence database. DDA Analysis: Processed with MaxQuant software. DIA Analysis: Processed using Spectronaut 17 software. Lysine crotonylation was included as a variable modification. A false discovery rate (FDR) threshold of 1 % was applied to ensure reliable protein identification.

### Western blot analysis and competition experiments

Protein samples (30 μg per lane) were resolved by 12 % SDS-PAGE and transferred to PVDF membranes. Membranes were blocked with 5 % skim milk for 1 h at room temperature and incubated overnight at 4 °C with primary antibodies targeting pan Kcr, Kac, Kpr, Kbu, Khib, Kmal, Kla, or Kbhb modifications (PTM Bio). Following incubation with secondary antibodies, membranes were washed and developed using the Clarity Western ECL substrate. Protein bands were visualized using the Tanon 5200-Multi imaging system.

To perform the competition experiments, the commercially available crotonyl pan-antibody was diluted at a ratio of 1:2000. The antibody solution was then incubated with either Boc-Lys(acetylation)-AMC or Boc-Lys(crotonylation)-AMC peptides (Guoping Pharmaceutical Co., LTD) at a final concentration of 1.35 µM, using an equivalent amount of non-acylated peptides as a control. The mixture was reacted at room temperature for 30 min to deplete the antibodies, followed by centrifugation at 10,000 rpm for 10 min to collect the supernatant. Subsequently, 15 µg of *S.pn* whole-cell proteins were separated by electrophoresis and transferred onto a PVDF membrane. After blocking with 5 % skim milk, the membrane was probed with the pre-treated antibody supernatant as the primary antibody. Following washing, a mouse secondary antibody was applied, and the signals were detected by chemiluminescence.

### Oligomerization analysis

Ply protein and its mutants (1 µg) were incubated with 125 µM crotonyl-CoA at 25 °C for 16 h. The reaction mixtures were then treated with 5 × SDS loading buffer (without β-mercaptoethanol) and further incubated at 55 °C for 10 min. Proteins were separated on 6 % SDS-PAGE gels and transferred to PVDF membranes. Oligomerization states were analyzed by Western blotting using anti-Ply antibodies.

### In vitro crotonylation assay

To evaluate the catalytic activity of SPD_0839 in vitro, crotonylation reactions were carried out using a reaction mixture consisting of 3 μg of SPD_0839 protein, 1.5 μg of purified substrate, 125 μM CrCoA, and a total reaction volume of 20 μL in a buffer solution (50 mM Tris HCl, pH 8.0, 1 mM DTT, 10 mM sodium butyrate, 10 % glycerol). The reaction mixture was subjected to incubation at a temperature of 30 °C for a duration of 4 h within a metal bath container. The addition of the sample loading buffer was performed to stop the reaction, and the Kcr level was assessed using western blotting. Separate gels were utilized to quantitatively monitor the loading of samples.

### Nanoparticle tracking analysis (NTA)

The NanoSight NS300 analyzer (Malvern, Shanghai, China) was used to measure particle size and concentration. The instrument was equipped with a 488 nm blue laser and a sCMOS camera. In a nutshell, samples were fed into the device after being diluted with PBS. Videos of three randomly selected viewpoints were recorded for each sample, with the following instrumental parameters set: temperature controller turned on; 25 °C temperature; 14 camera level; 60-second capture time. The parameters used to analyze the videos were as follows: detection threshold of three; viscosity of water (0.89 cP). The software NanoSight NTA 3.4 (Malvern) was used to provide the particle size and mean concentration that were calculated from the three videos.

### Bioinformatics analysis

Bioinformatics analyses were performed to interpret protein interaction networks and functional enrichment. Pathway and motif analysis was conducted using the Wu Kong platform (https://www.omicsolution.com/wkomics/main/), which provided insights into Kyoto Encyclopedia of Genes and Genomes (KEGG) pathways and sequence motifs. Protein-protein Interactions were predicted using the STRING database. The network was analyzed with the Molecular Complex Detection (MCODE) module in Cytoscape to identify functional clusters. Biological processes, molecular functions, and cellular composition were annotated using Cytoscape.

### Statistical analysis

All in vitro experiments were conducted in triplicate to ensure reproducibility. Results are expressed as means ± standard error of the mean (SEM). Statistical significance was assessed using appropriate tests, with significance thresholds defined as follows: P < 0.05: Significant (*), P < 0.01: Highly significant (**), P < 0.001: Extremely significant (***), P < 0.0001: Extremely significant (****), NS, no significance.

## Data availability

The raw proteomic data and search results have been deposited to the ProteomeXchange Consortium via the PRIDE [32] partner repository on December 14, 2024 and can be accessed with the reviewer account at https://www.ebi.ac.uk/pride under accession no. PXD058867, username: reviewer_pxd058867@ebi.ac.uk, Password: 9MrrFgIOz6rS.

## Ethics statement

All experimental protocols involving animals were reviewed and approved by the Ethics Committee for Animal Experiments at Jinan University (IACUC Approval No. 2021102925-06). The study was conducted in strict compliance with the institutional guidelines for the care and use of laboratory animals.

## Results

### Kcr regulates the virulence and adaptive survival of clinical multidrug-resistant *S.pn*

**Fig. 1:**
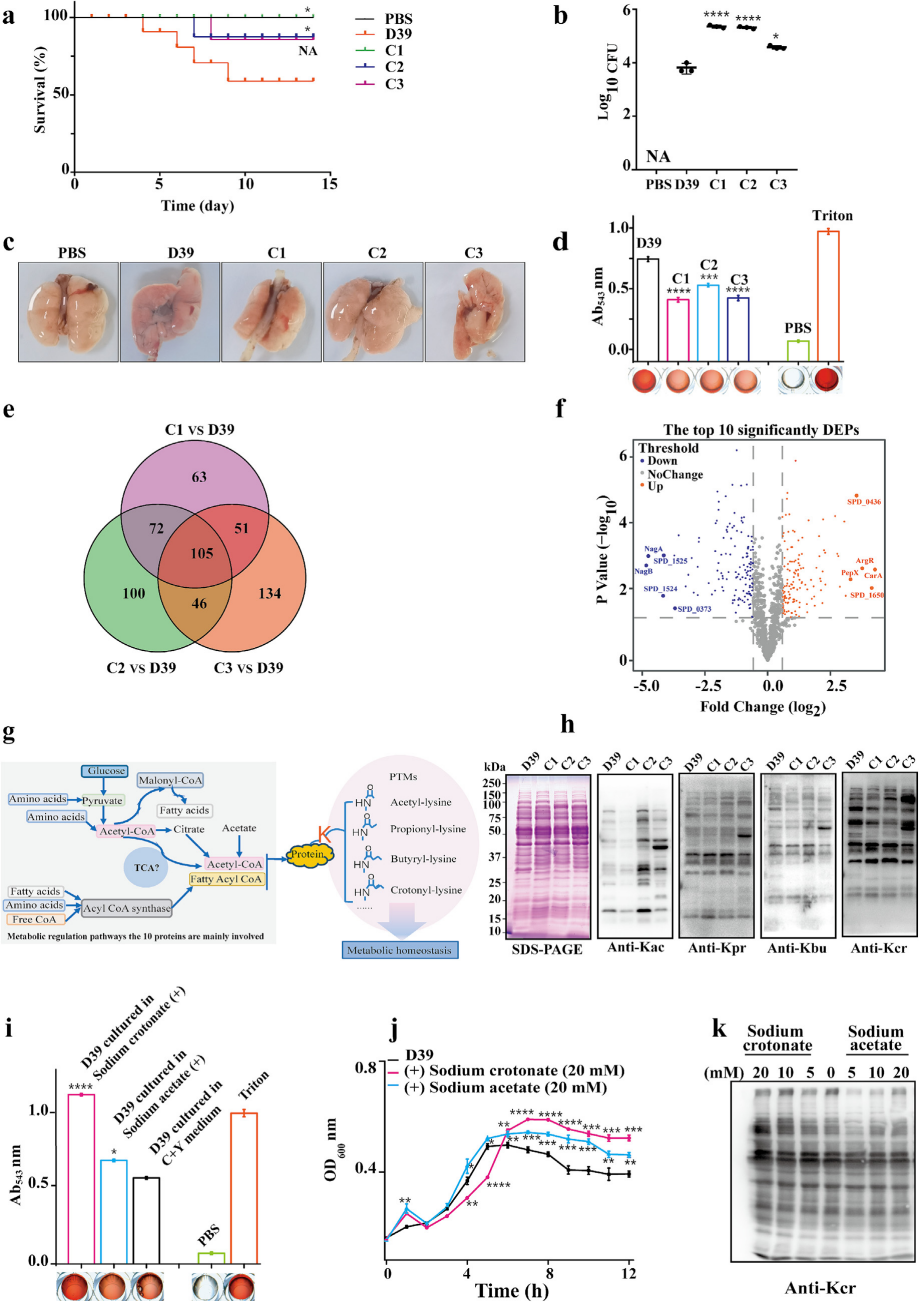
Pneumococcal virulence and proteome profiling in clinical *S.pn* strains compared to D39. a. Kaplan–Meier survival curves of mice intranasally infected with D39 or three clinically isolated strains (*n* = 5 per group). b. Bacterial load in lung tissue (colony-forming units, CFU) 36 h post-infection with D39 or clinical isolates. c. Gross pathology of lungs from mice infected with D39 or clinical strains, with PBS serving as a negative control. d. Hemolytic activity of D39 and clinical isolates, visualized using a microplate assay. e. Venn diagram showing differentially expressed proteins (DEPs) among clinical isolates compared to D39. f. Volcano plot highlighting the top five upregulated and downregulated proteins among DEPs. g. Schematic representation of central carbon metabolism, illustrating acyl-CoAs and lysine post-translational modifications (PTMs) in *S.pn*. h. Western blot analysis comparing lysine acylation levels (including crotonylation) in whole-cell lysates of D39 and clinical strains. i. Hemolytic activity of D39 cultured under various conditions. j. Growth curves of D39 in C + Y medium supplemented with different substrates. Optical density was measured at 600 nm (OD_600_). k. Western blot analysis of crotonylation levels in whole-cell lysates of D39 cultured under different conditions using a pan-crotonylation antibody. In Figure b, d, i, n = 3, and Data are mean ± standard deviation (s. d). One-way ANOVA with Dunnett's multiple comparisons test. In Figure j, Unpaired student’s two-tailed *t*-test was applied to compare two experimental groups. Statistical significance was defined as *p < 0.05, **p < 0.01, ***p < 0.001, ****p < 0.0001, NS, no significance.

To investigate the biological determinants of adaptive survival of clinical bacteria, we used three clinical multidrug-resistant *S.pn* strains and the laboratory strain D39 to intranasally infect mice (16S rRNA similarity data for the bacterial strains is listed in Supplementary Table 1). Survival rates were monitored over two weeks. Animal experiments showed that mice infected with the clinical *S.pn* strains exhibited significantly lower mortality rates compared to those infected with *S.pn* D39 (Fig. 1a, 1c). However, bacterial loads in the lungs of mice infected with clinical strains were significantly higher than in those infected with *S.pn* D39 (Fig. 1b). Additionally, hemolysis assays revealed that clinical multidrug-resistant *S.pn* strains exhibited markedly lower hemolytic activity compared to *S.pn* D39 (Fig. 1d). These findings suggest that clinical strains exhibit high invasiveness but reduced virulence. This phenomenon aligns with observations of MT93 (Metabolic genotypes 93), a newly isolated strain of the common *S.pn* serotype 23B, which exhibits reduced hemolysis compared to MT23 (Metabolic genotypes 23 of serotype *S.pn* 23B) but shows greater efficacy in invading nasopharyngeal cells [33].

To uncover different virulence mechanisms between the laboratory strain *S.pn D39* and clinical *S.pn* strains, DIA-based quantitative proteomics was performed. A total of 105 differentially expressed proteins (DEPs) were identified in all three clinical strains (fold change > 1.5) (Fig. 1e). Among these, we focused on the top five upregulated proteins (ArgR, PepX, CarA, SPD_0436, and SPD_1650) and the top five downregulated proteins (NagA, NagB, SPD_1524, SPD_1525, SPD_0373) (Fig. 1f). Except for SPD_1650, an iron-binding protein, most of these proteins are key enzymes in carbon metabolism. Carbohydrate metabolism is known to play a pivotal role in pneumococcal adaptation to ecological niches and infection progression [34,35]. The expanding “metabolic genotype” (MT) includes auxiliary genes associated with virulence and antimicrobial resistance (AMR) [33]. Central carbon metabolism generates various acyl-CoAs, which serve as precursors for post-translational modifications (PTMs) such as acetylation (Kac), propionylation (Kpr), butyrylation (Kbu), and crotonylation (Kcr). These reversible PTMs will rapidly fine-tune protein abundance or activity in many important biological pathways (Fig. 1g).

Western blotting with pan-antibodies for various PTMs revealed significant changes in Kac and Kcr levels in clinical *S.pn* strains compared to *S.pn D39* (Fig. 1h). To evaluate the effects of PTMs on *S.pn* virulence and growth, we supplemented the culture medium with sodium acetate and sodium crotonate, as these short-chain fatty acid salts can serve as acyl-CoA precursors[26]. Hemolysis assays showed that sodium crotonate significantly enhanced hemolytic activity, while sodium acetate caused only a slight increase (Fig. 1i). Growth curve analysis revealed that sodium acetate had minimal effects on bacterial growth, whereas sodium crotonate inhibited growth during the logarithmic phase. This aligns with the observed slower growth of clinical isolates compared to *S.pn* D39 (Fig. 1j, Supplementary Fig. 1a–b). Western blotting further showed that sodium crotonate supplementation significantly increased Kcr levels, with minimal effects on Kac levels, while sodium acetate had no effect on Kcr (Fig. 1k, Supplementary Fig. 1c). Together, these results demonstrate that Kcr is involved in regulating the virulence, growth, and adaptive survival of clinically isolated *S.pn*.

### SPD_0839 is a novel crotonyltransferase in *S.pn*

The above findings suggest that Kcr plays a significant role in *S.pn* growth and virulence. Recent studies have shown that Kcr is widely distributed in various bacterial species and plays a key role in regulating metabolism, virulence, and adaptation to intracellular environments [26,28,30,36]. Identifying the crotonyltransferase responsible for Kcr is thus critical for understanding its regulatory mechanisms in *S.pn*. Given that many identified bacterial acetyltransferases (KATs) belong to the GNAT family and often exhibit multifunctional acyltransferase activity [37,38], we hypothesized that GNAT-family proteins in *S.pn* might also possess crotonyltransferase (KCT) activity. Although GNAT proteins exhibit limited sequence homology across species, they share a conserved core domain comprising six or seven β-strands and four α-helices, with motif A being the most conserved (Supplementary Fig. 1d).

**Fig. 2:**
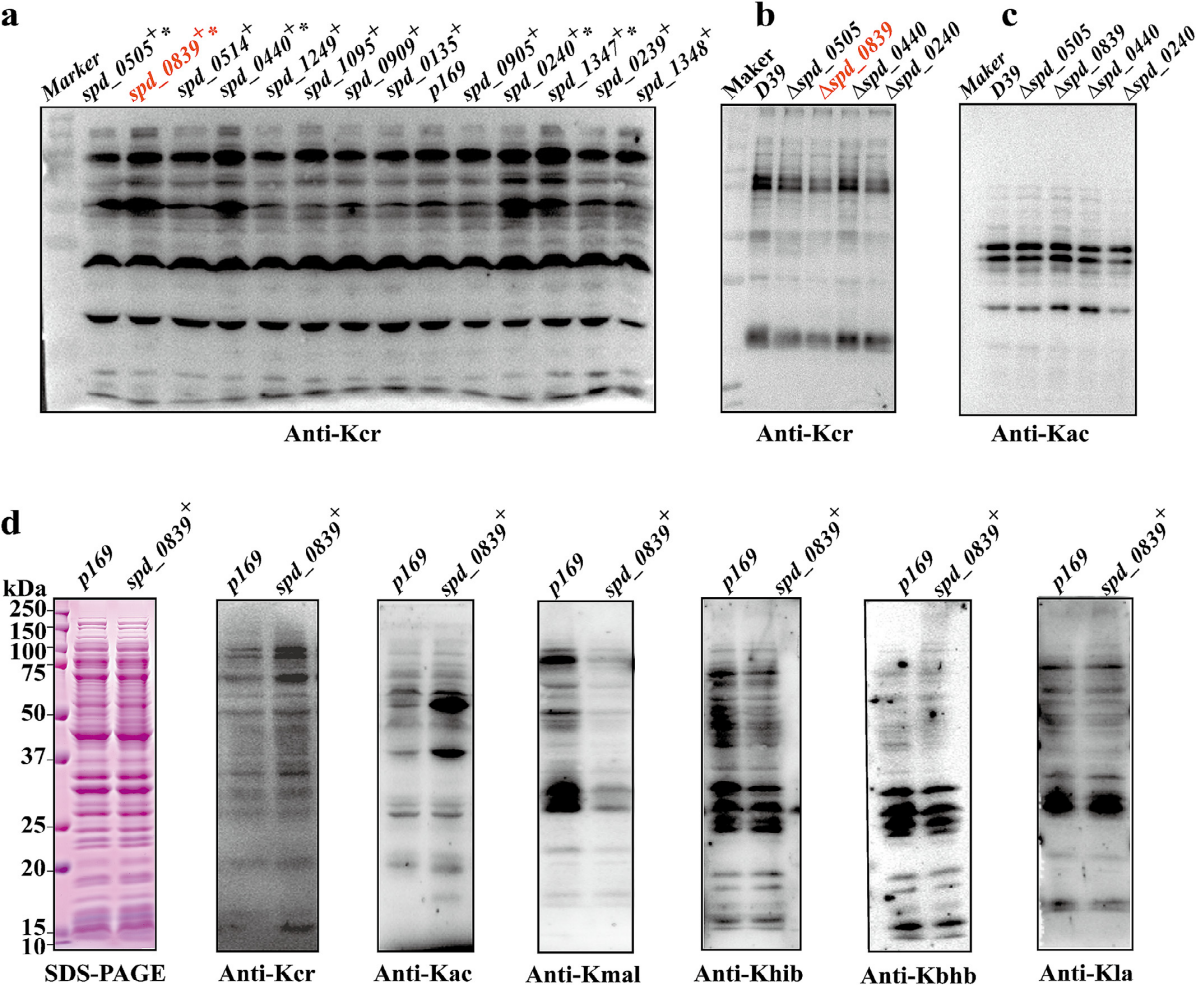
Identification of SPD_0839 as a lysine crotonyltransferase. a. Western blot analysis of crotonylation (Kcr) levels in whole-cell lysates from strains overexpressing SPD_0839, SPD_0505, SPD_0440, SPD_0240, and SPD_1347. D39 with an empty plasmid (p169) served as a control. b, c. Western blot detection of Kcr and acetylation (Kac) levels in whole-cell lysates of knockout strains (Δ*spd_0839*, Δ*spd_0505*, Δ*spd_0440*, and Δ*spd_0240*) compared to the wild-type D39 strain. d. Western blot analysis of lysine acylation levels in whole-cell lysates from *spd_0839^+^*.

To investigate whether GNAT-family proteins in *S.pn* act as crotonyltransferases, we constructed 13 overexpression plasmids encoding GNAT-family proteins containing the core structural domains and introduced them into *S.pn D39*. Western blotting revealed that several overexpressing strains (named *spd_0839^+^*, *spd_0505^+^*, *spd_0440^+^*, *spd_0240^+^*, and *spd_1347^+^*) exhibited significantly increased Kcr levels compared to control strain with empty plasmid (p169) (Fig. 2a, Supplementary Fig. 1e).

To confirm the role of these five GNAT-family proteins in Kcr regulation, we generated knockout strains (Δ*spd_0839*, Δ*spd_0505*, Δ*spd_0440*, and Δ*spd_0240*). We were unable to knock out *spd_1347* in *S.pn* D39, likely due to its essential role in bacterial viability. Among these mutants, Δ*spd_0839* showed significantly reduced Kcr levels compared to *S.pn* D39, while the other mutants exhibited no changes. Notably, Δ*spd_0839* had no effect on Kac levels (Fig. 2b–c). We also generated a recombinant antibody specific to SPD_0839 and confirmed its expression in Δ*spd_0839* and *spd_0839*^+^ strains for subsequent functional studies. Consistent with the Kcr levels in clinical *S.pn* strains, SPD_0839 showed low expression in all clinical strains (Supplementary Fig. 1f–g). Competition experiments validated the specificity and affinity of the Kcr antibody (Supplementary Fig. 1h). These results suggest that SPD_0839 has a global regulatory role in Kcr of *S.pn* proteome, making it the primary candidate crotonyltransferase for further Kcr investigation.

Given that structural variations in GNAT-family proteins enable them to accommodate diverse acyl-CoAs, we tested whether SPD_0839 functions as a multifunctional acyltransferase by using pan-antibodies targeting lysine crotonylation (Kcr), acetylation (Kac), malonylation (Kmal), 2-hydroxyisobutyrylation (Khib), β-hydroxybutyrylation (Kbhb), and lactylation (Kla). Overexpression of SPD_0839 caused a global increase in Kcr levels across whole molecular weights, with minimal changes in Kac levels and no changes in Kbhb or Kla. In contrast, Kmal and Khib levels decreased significantly (Fig. 2d). These findings imply potential crosstalk between PTMs regulated by SPD_0839 and confirm its role as a major crotonyltransferase in *S.pn*.

### Phe93 is crucial for CrCoA binding and affects Kcr catalysis capability of SPD_0839

**Fig. 3:**
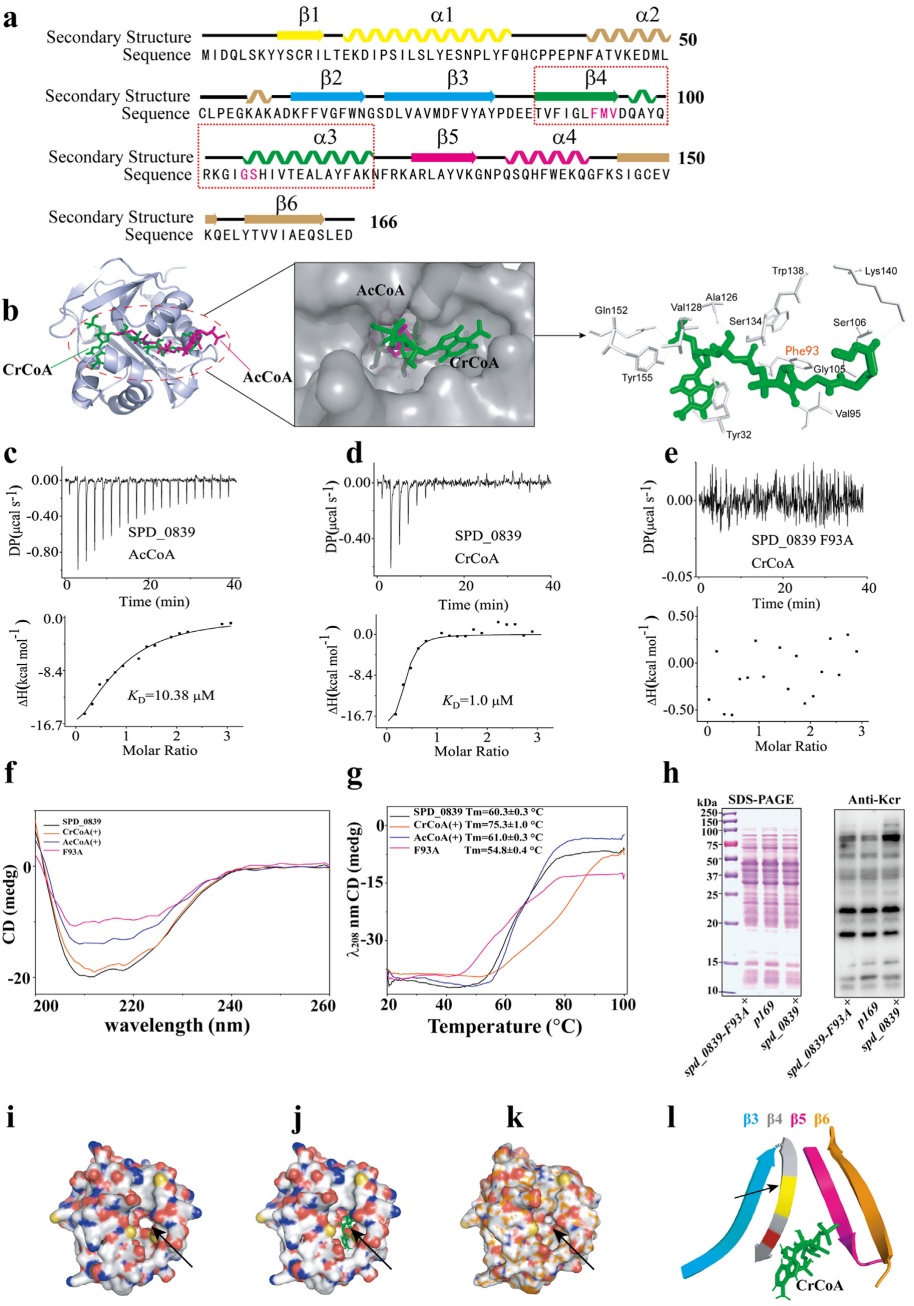
Phe93 is a critical binding site in SPD_0839 for CrCoA. a. Structural prediction of the GNAT core domain of SPD_0839. Predicted secondary structures are shown, with β-sheets as arrows, α-helices as ribbons, and the conserved motif-A enclosed in a red frame. b. Molecular docking results showing CrCoA (green stick) and AcCoA (purple stick) bound to SPD_0839. c-e. Isothermal titration calorimetry (ITC) analysis demonstrating the binding affinity of wild-type SPD_0839 and the F93A mutant with AcCoA and CrCoA. f-g. Circular dichroism (CD) analysis of conformational and thermal stability changes in SPD_0839 bound to AcCoA or CrCoA. h. Western blot detection of Kcr levels in whole-cell lysates from strains *spd_0839^+^* and *spd_0839^+^-*F93A, with *p169* as a control. i-k. Electron density maps showing structural features of SPD_0839, SPD_0839 bound to CrCoA, and SPD_0839-F93A. Arrows highlight the hydrophobic pocket. l. V-shaped opening formed by β4 and β5 strands in SPD_0839, with Phe93 marked in red and the β-bulge highlighted in yellow. (For interpretation of the references to color in this figure legend, the reader is referred to the web version of this article.)

To investigate the CrCoA and AcCoA binding pocket of SPD_0839, we first analyzed its GNAT core domain. The conserved structure consists of six β-strands and four α-helices connected in the order β1, α1, α2, β2, β3, β4, α3, β5, α4, β6 (Fig. 3a). Molecular docking using the SPD_0839 structure predicted by AlphaFold2 revealed that both CrCoA and AcCoA bind to a shared pocket. However, CrCoA showed a lower free binding energy (−9.1 kcal/mol) compared to AcCoA (−8.6 kcal/mol), indicating a stronger and more specific interaction. CrCoA occupied the hydrophobic cavity in a closely packed manner, suggesting a preference for CrCoA binding (Fig. 3b, Supplementary Fig. 2a).

To further assess the binding affinity of SPD_0839 for CrCoA, we performed Isothermal Titration Calorimetry (ITC). Consistent with docking results, SPD_0839 exhibited a binding affinity (KD) of 1 μM for CrCoA, an order of magnitude higher than its KD for AcCoA (10.38 μM) (Fig. 3c–d). Using Circular Dichroism (CD) spectroscopy, we investigated the structural and thermal stability changes in SPD_0839 upon binding CrCoA and AcCoA. While secondary structures remained largely unaffected, CrCoA binding significantly enhanced thermal stability, increasing the melting temperature (Tm) by 15 °C. In contrast, AcCoA binding caused only a 1 °C increase in Tm (Fig. 3f–g). Together, these results confirm that SPD_0839 prefer to binding CrCoA, enabling it to effectively catalyze Kcr.

To further study catalytic activity of SPD_0839, we performed a detailed structural analysis to identify the crucial CrCoA-binding residues in SPD_0839. Docking results highlighted a hydrophobic pocket composed of 12 residues. Among these, 11 residues (Tyr32, Phe93, Gly105, Ser106, Ala126, Val128, Ser134, Trp138, Lys140, Gln152, and Tyr155) form hydrogen bonds with CrCoA, while Val95 contributes through hydrophobic interactions (Fig. 3b). Notably, Phe93, Met94, Val95, Gly105, and Ser106, located in the conserved motif A of GNAT proteins, form the AcCoA binding pocket. Moreover, all five residues interact with AcCoA, though Met94 does not interact with CrCoA (Fig. 3a, Supplementary Fig. 2b).

To assess the functional roles of these key residues, we generated alanine mutants (F93A, M94A, V95A, G105A, and S106A) and purified the resulting proteins. ITC measurements showed that mutations in Val95, Gly105, and Ser106 significantly reduced CrCoA binding affinity, while M94A retained binding ability comparable to the wild-type protein (Supplementary Fig. 3a–d). Strikingly, the F93A mutation abolished CrCoA binding entirely (Fig. 3e). Mutations combining F93A-M94A-V95A in β4 or G105A-S106A in α3 also resulted in a complete loss of CrCoA binding (Supplementary Fig. 3e–f). Thermal stability assays revealed that all mutant proteins exhibited reduced stability compared to the wild-type protein, with F93A showing the most pronounced decrease—a 20 °C reduction in Tm (Fig. 3f-g, Supplementary Fig. 3g–h). These results indicate that Phe93 plays a critical role in CrCoA binding and protein stability. Further structural analysis showed that the hydrophobic cavity accommodating CrCoA collapsed in the F93A mutant, as alanine substitution disrupted the pocket's shape (Fig. 3i–k). Phe93, located in the β4 strand of motif A, forms a V-shaped opening parallel to the β5 strand, a configuration critical for CrCoA binding and catalytic activity, as seen in other GNAT proteins[39] (Fig. 3l).

To confirm the role of Phe93 in Kcr catalysis in vivo, we constructed an overexpression strain, *spd_0839*-F93A^+^, and compared it to the wild-type overexpression strain, *spd_0839*- wt, and the control strain with empty plasmid, *p169*. Western blot analysis showed that *spd_0839*- wt exhibited increased Kcr levels compared to *p169*, while *spd_0839*-F93A^+^ showed no significant changes (Fig. 3h). In summary, Phe93 is a critical residue for CrCoA binding in SPD_0839, influencing its structural stability and Kcr catalytic activity in both in vitro and in vivo.

### Crotonyltransferase SPD_0839 negatively regulates *S.pn* growth and virulence

**Fig. 4:**
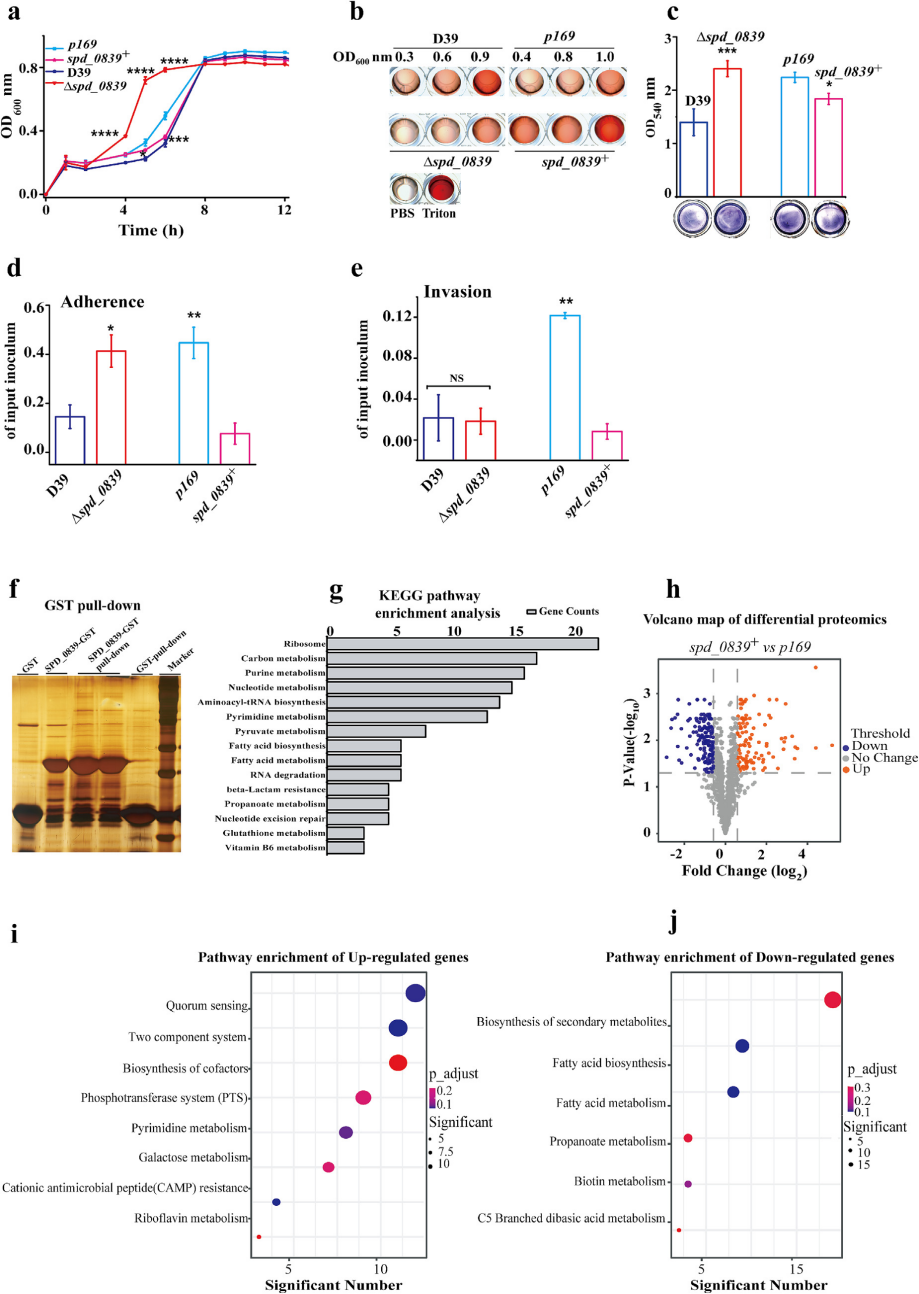
SPD_0839 influences bacterial growth and virulence. a. Growth curves of Δ*spd_0839* and *spd_0839^+^* strains. Optical density at 600 nm (OD_600_) indicates bacterial growth. b. Hemolytic activity of D39, Δ*spd_0839*, and *spd_0839^+^*, presented in a microplate assay. c. Quantification of biofilm formation using 1 % crystal violet staining. Absorbance at 540 nm was measured to assess biofilm density. d-e. Adhesion and invasion capabilities of Δ*spd_0839* and *spd_0839^+^* strains evaluated using human A549 lung epithelial cells. f. Silver staining of GST-SPD_0839 pull-down assay showing potential interacting proteins. g. KEGG pathway enrichment analysis of proteins identified in the GST-SPD_0839 pull-down experiment. h. Volcano plot displaying differential proteins in *spd_0839^+^* compared to control strain (*p169*). i-j. KEGG pathway enrichment analysis of upregulated (i) and downregulated (j) proteins in *spd_0839^+^*. In Figure a, c-e, n = 3, and Data are mean ± standard deviation (s. d). Unpaired student’s two-tailed *t*-test was applied to compare two experimental groups. Statistical significance was defined as *p < 0.05, **p < 0.01, ***p < 0.001, ****p < 0.0001, NS, no significance. (For interpretation of the references to color in this figure legend, the reader is referred to the web version of this article.)

Recent studies have demonstrated that crotonylation plays a critical role in regulating spindle involvement during cell division in vertebrates [40]. This led us to hypothesize that SPD_0839 may influence bacterial growth by mediating crotonylation. To test this, we measured the growth curves of the Δ*spd_0839* and *spd_0839*^+^ strains. Interestingly, the growth rate of Δ*spd_0839* reaching the plateau phase 5–6 h after inoculation was significantly faster than that of the wild-type *S.pn* D39 strain*.* In contrast, D39, *p169* (vector control), and *spd_0839*^+^ strains required approximately 8 h to reach the same phase. However, during the logarithmic growth phase, *spd_0839*^+^ grew more slowly than the *p169* control (Fig. 4a). These results suggest that SPD_0839 negatively regulates *S.pn* growth by modulating Kcr.

During infection, *S.pn* produces several adhesion and virulence factors, including pneumolysin (PLY), choline-binding proteins PspA and PspC, and neuraminidase NanA, which facilitate bacterial colonization of the mucosal surfaces in the host’s nasopharynx and upper respiratory tract. Among these, PLY is a critical virulence factor that contributes to inflammation and tissue penetration by forming pores in host cells and blocking complement activation. PLY also plays a key role in pneumococcal meningitis by enabling *S.pn* to cross the blood–brain barrier. To evaluate the effects of SPD_0839 on virulence, we analyzed hemolysis, biofilm formation, adhesion and invasion. We found that the hemolytic activity of Δ*spd_0839* was significantly reduced at all growth stages compared to D39, whereas *spd_0839*^+^ exhibited enhanced hemolysis (Fig. 4b). Biofilm formation was notably increased in Δ*spd_0839* compared to D39 (Fig. 4c). Previous findings that *S.pn* strains with thickened biofilms demonstrate increased adhesion but reduced invasiveness [41]. To further investigate adhesion and invasion, we used human lung epithelial A549 cells as a host model. Adherence to A549 cells was significantly increased in Δ*spd_0839* but decreased in *spd_0839*^+^, consistent with the biofilm and growth results. However, the knockout of *spd_0839* had no significant effect on bacterial invasion ability, while overexpression of SPD_0839 led to a marked reduction in invasion capability (Fig. 4d–e). Together, these results demonstrate that SPD_0839 modulates *S.pn* growth, biofilm formation, adhesion, and hemolytic activity, exerting a broad influence on bacterial virulence.

To gain deeper insights into the biological functions and regulatory network of SPD_0839, we performed GST pull-down assays combined with mass spectrometry (MS). A total of 342 interacting proteins were identified as potential SPD_0839 binding partners. KEGG enrichment analysis revealed that many of these proteins are involved in key metabolic pathways, including carbon metabolism, nucleotide metabolism, fatty acid metabolism, purine and pyrimidine metabolism, and β-lactam resistance (Fig. 4f–g).

To further elucidate the pathways regulated by SPD_0839, we conducted DIA-based quantitative proteomics on D39 and *spd_0839*^+^ strains. A total of 141 upregulated proteins and 106 downregulated proteins were identified (fold change > 1.5) (Fig. 4h). Upregulated proteins were primarily enriched in pathways such as the PTS system, pyrimidine metabolism, riboflavin metabolism, and galactose metabolism. Conversely, downregulated proteins were associated with pathways such as secondary metabolite biosynthesis, fatty acid metabolism, and propionate metabolism (Fig. 4i–j).

These results suggest that SPD_0839-mediated Kcr affects numerous critical metabolic pathways in *S.pn*. By modulating Kcr on a wide range of proteins, SPD_0839 has the potential to regulate metabolism and biosynthesis of macromolecules, thus influencing bacterial growth, virulence, and adaptation.

### Key endogenous substrate proteins of SPD_0839 involved in metabolism and virulence

**Fig. 5:**
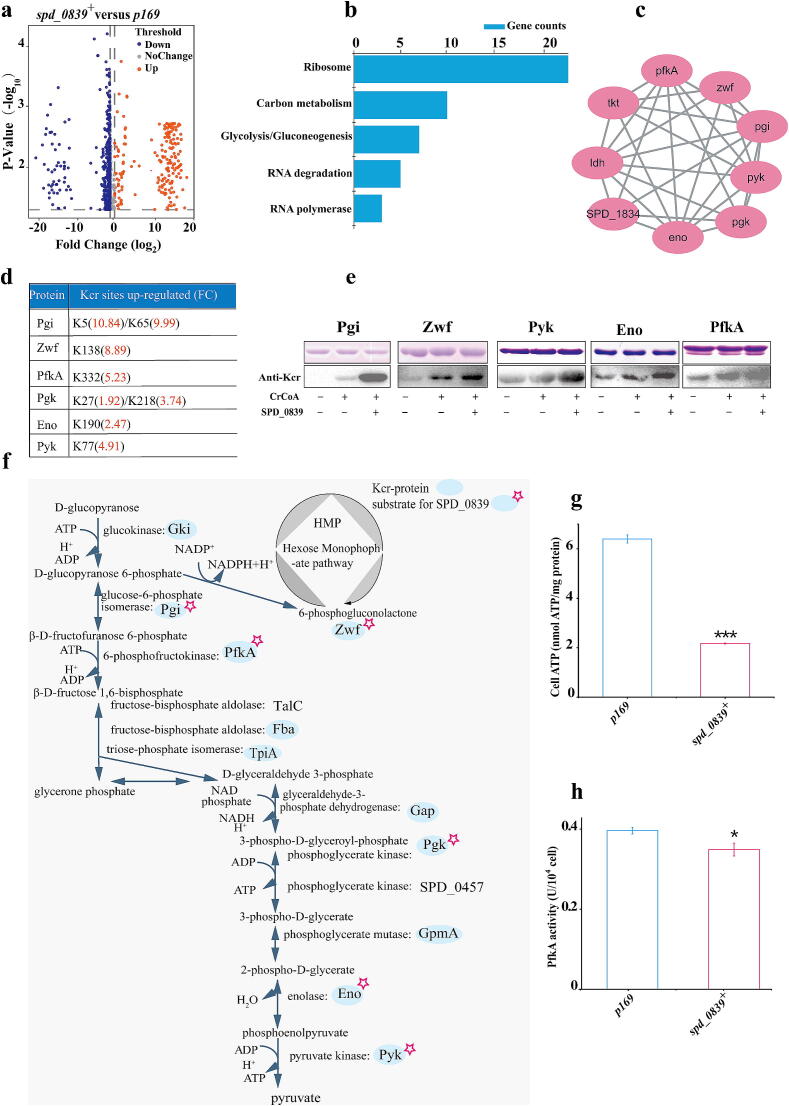
Endogenous substrates targeted by SPD_0839 for Kcr modification. a. Volcano plot of Kcr peptide ratios in *spd_0839^+^* versus *p169*, normalized to protein abundance. b. KEGG pathway enrichment analysis of proteins with increased Kcr sites. c. Protein-protein interaction (PPI) network showing upregulated Kcr sites on proteins involved in energy metabolism pathways. d. Overview of upregulated Kcr sites in enzymes central to carbon metabolism. e. In vitro analysis of SPD_0839-mediated Kcr modification in key enzymes, including Pgi, Zwf, Eno, Pyk, and PfkA. f. Schematic diagram illustrating crotonylation of central carbon metabolic enzymes catalyzed by SPD_0839. g, h. Intracellular ATP content (g) and PfkA enzyme activity (h) in *spd_0839^+^*, showing SPD_0839-mediated regulation. In Figure g-h, n = 3, and Data are mean ± standard deviation (s. d). Unpaired student’s two-tailed *t*-test was applied to compare two experimental groups. Statistical significance was defined as *p < 0.05, **p < 0.01, ***p < 0.001, ****p < 0.0001, NS, no significance.

To further reveal the biological function of SPD_0839, we identified endogenous substrates and established its regulatory network via quantitative Kcr proteomic analysis on the *spd_0839*^+^ and *p169* strains. Quantification data indicated that SPD_0839 overexpression results in upregulation of Kcr levels at 205 sites across 153 proteins, suggesting that SPD_0839 modulates their crotonylation either directly or indirectly (Fig. 5a). Notably, the proteins regulated by SPD_0839 are involved in essential biological processes, including ribosome assembly, carbon metabolism, glycolysis/gluconeogenesis, RNA degradation, and RNA polymerase activity (Fig. 5b). Protein-protein interaction (PPI) network analysis demonstrated that the potential substrate proteins predominantly cluster into functional groups associated with ribosome biogenesis, energy metabolism, and protein synthesis (Fig. 5c).

Next, we focused on the regulatory effect of SPD_0839 on enzymes associated with energy metabolism, which were significantly upregulated in the *spd_0839*^+^ strain (Fig. 5d). To investigate SPD_0839′s catalytic activity, purified Pgi, Zwf, PfkA, Pgk, Eno and Pyk enzymes were subjected to in vitro acylation reactions. Results showed that SPD_0839 successfully transferred crotonyl groups from CrCoA to these enzymes, increasing their Kcr levels to varying degrees (Fig. 5e).

Given the potential functional impact of Kcr on these enzymes, we hypothesized that Kcr mediated by SPD_0839 might influence intracellular ATP production. This is particularly relevant for PfkA, a key rate-limiting enzyme in glycolysis, which catalyzes the conversion of fructose-6-phosphate to fructose-1,6-bisphosphate. To explore this, we monitored changes in PfkA activity following Kcr mediated by SPD_0839. As expected, SPD_0839 overexpression leads to reduced intracellular PfkA activity, which in turn resulted in a substantial decrease in bacterial ATP levels (Fig. 5f–h). These findings highlight the role of SPD_0839 in modulating the functions of endogenous substrates through Kcr, ultimately affecting intracellular energy metabolism.

### SPD_0839 regulates *S.pn* hemolysis by mediating PLY crotonylation

In our preceding study, we observed that manipulation of SPD_0839, either through knockout or overexpression, alters the hemolytic activity of PLY in *S.pn* (Fig. 4b). Notably, Kcr levels at PLY residues K171 and K442 were upregulated in the presence of SPD_0839 (Supplementary Fig. 4a and b), suggesting key Kcr regulatory role in hemolytic activity.

**Fig. 6:**
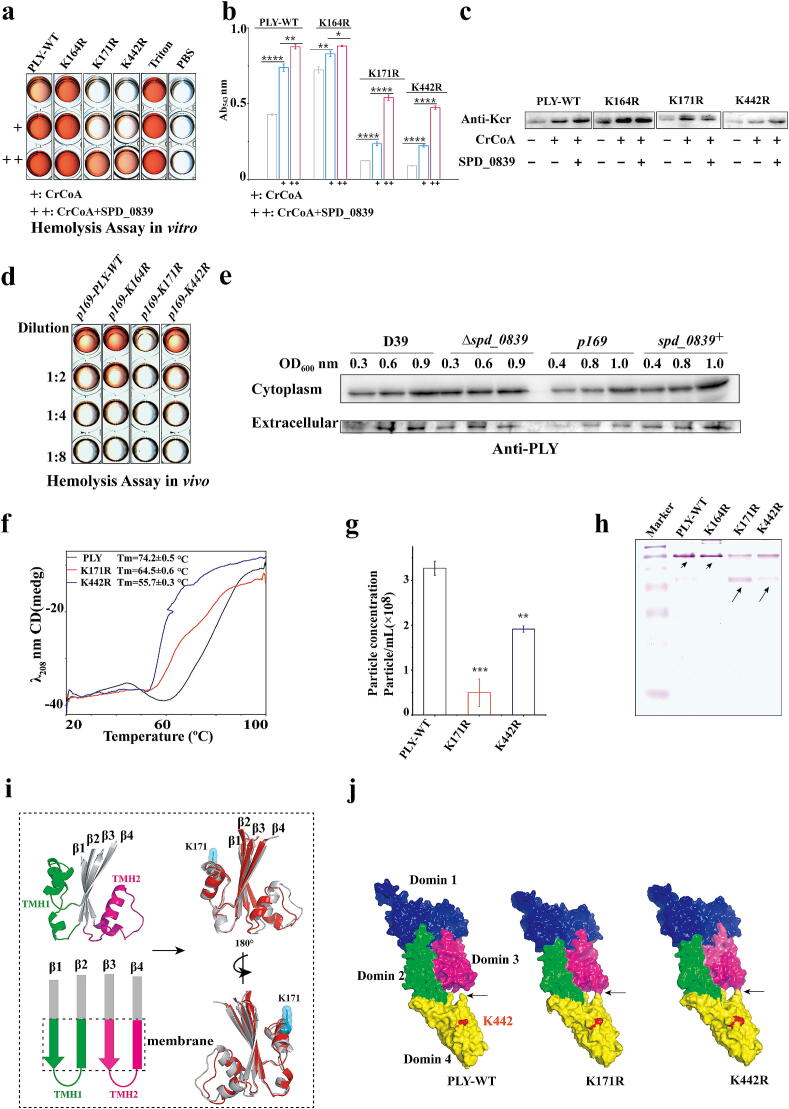
SPD_0839 enhances hemolytic activity of PLY by regulating Kcr at K171 and K442. a-b. In vitro hemolysis assays showing the hemolytic activity of wild-type (WT) and mutant PLY (K-to-R substitutions). c. Western blot analysis of Kcr levels in WT and mutant PLY catalyzed by SPD_0839. d. Hemolytic activity of PLY released into the culture medium by D39 strains overexpressing WT or mutant PLY (K-to-R substitutions). e. Expression levels of intracellular and extracellular PLY in Δ*spd_0839* and *spd_0839^+^*, using D39 and *p169* as controls. f. Thermal stability of PLY and mutants (K171R and K442R) analyzed via circular dichroism (CD). g. Particle concentration of PLY and mutants (K171R, K442R) measured using nanoparticle tracking analysis. h. Native gel analysis of PLY and its mutants (K171R, K442R). Short arrows indicate oligomeric forms, while long arrows indicate monomeric forms. i. Structural changes in TMH1 and TMH2 induced by K171R mutation. K171 is shown in cyan (sphere and stick representation). The domain 3 structure of WT PLY is in gray, while K171R mutant is in red. j. Electron density maps of WT PLY and K171R or K442R mutants. Domains 1–4 are color-coded, with K442 in domain 4 highlighted in red. Arrows indicate conformational changes at the junction of domains 3 and 4 caused by mutations. In Figure b, g, n = 3, and Data are mean ± standard deviation (s. d). One-way ANOVA with Dunnett's multiple comparisons test. Statistical significance was defined as *p < 0.05, **p < 0.01, ***p < 0.001, ****p < 0.0001, NS, no significance. (For interpretation of the references to color in this figure legend, the reader is referred to the web version of this article.)

To confirm that SPD_0839 catalyzes PLY crotonylation, in vitro crotonylation assays were performed using purified wild-type PLY and mutant proteins in which specific lysine residues (K164R, K171R and K442R) were substituted with arginine. This substitution was chosen to mimic the de-crotonylated state due to arginine's charge similarity to lysine (Supplementary Fig. 5a). Hemolysis experiments and western blot analysis demonstrated that SPD_0839 catalyzed crotonylation of PLY, enhancing hemolytic activity, while K171R and K442R nearly lost hemolytic activity entirely (Fig. 6a–c). Further analysis revealed that co-incubation with CrCoA did not restore hemolytic activity in K171R and K442R mutant. The significantly reduced Kcr levels and hemolytic capacity observed in K171R and K442R mutants confirm that crotonylation at these sites is critical for PLY function. To assess the in vivo impact of PLY crotonylation on hemolysis, we constructed strains expressing p169-K164R, p169-K424R, and p169-K442R mutants. Results aligned with in vitro findings, with p169-K171R showing significantly diminished hemolytic capacity in vivo (Fig. 6d).

As PTMs are known to influence protein stability, we assessed the effects of Kcr on PLY structure and thermal stability. CD analysis revealed no significant differences in secondary structure composition between wild-type PLY and its mutants (K171R and K442R). However, thermal denaturation assays showed significant reductions in melting temperatures (Tm) for K171R (64.5 °C) and K442R (55.7 °C), compared to wild-type PLY (74.2 °C), indicating decreased protein stability (Fig. 6f, Supplementary Fig. 5b). We found that PLY protein levels, both intracellular and secreted into the culture medium, are decreased following *spd_0839* knockout. Conversely, SPD_0839 overexpression significantly increases PLY amount (Fig. 6e). These results indicate that SPD_0839 enhances PLY stability via Kcr, which further promotes PLY oligomerization and hemolytic activity.

Subsequent nanoparticle tracking analysis showed that oligomer formation was significantly reduced in K171R and K442R mutants compared to wild-type PLY (Fig. 6g). Native gel electrophoresis confirmed these findings, showing an increase in monomer forms and reduced oligomerization for K171R and K442R relative to wild-type PLY and K164R (Fig. 6h).

Structure analysis revealed that crotonylation occurs in PLY domains 1, 3, and 4, but not in the “neck” domain 2. K171, located in domain 3, participates in the formation of transmembrane β-hairpins (TMHs), which are essential for PLY conformational changes and pore formation. In the K171R mutant, the TMH1 helix formation was disrupted, leading to altered conformations of both TMH1 and TMH2 (Fig. 6i). Lys442, located in domain 4, is critical for initial membrane recognition and binding. Structural analysis revealed that Lys442 forms a bulge on the PLY surface, facilitating insertion into the cholesterol membrane. This bulge disappears entirely in the K442R mutant, and K171R mutation further impacts the K442 bulge. Both K171R and K442R significantly altered the interactions between domains 3 and 4, which changed from a separated state to a bound form (Fig. 6j, Supplementary Fig. 5c). These conformational changes likely impair pore formation and hemolytic activity. In the previously characterized PLY pre-pore formation process, PLY dimers exhibit a distinct conformational arrangement (PDB entry 2BK2). So, we also used AlphaFold 3 to model the structures of K171R or K442R dimers. Structural analysis revealed that decrotonylation at either K171 or K442 induces dramatic changes in dimer conformation and spatial organization. PLY oligomerization assays consistently demonstrate that crotonylation promotes PLY oligomer formation and consequently enhances its hemolytic activity (Supplementary Fig. 6).

These findings demonstrate that SPD_0839-mediated crotonylation at K171 and K442 enhances PLY stability, promotes oligomerization, and facilitates pore formation, thereby increasing hemolytic activity. This regulatory mechanism underscores the critical role of SPD_0839 in *S.pn* virulence.

## Discussion

The World Health Organization recognizes antimicrobial resistance in pneumonia as a critical global health threat, demanding urgent identification of novel therapeutic targets and strategies, despite reported advancements in antimicrobial research methodologies across other bacterial specie [42,43]. Post-translational modifications (PTMs) are known to regulate key catalytic enzymes involved in numerous metabolic pathways, making them critical to bacterial adaptation and survival. Studies have established a strong correlation between PTMs and bacterial virulence, adaptability, and antibiotic resistance. For instance, our group found that deacetylation of pyruvate kinase PykF results in an increase of energy production, in turn, increases drug sensitivity of *E.coli*. Similarly, acetylation of Gapa and GPMA in *E. coli* inhibits enzymatic activity, reducing glycolysis [44], while H-NS acetylation in *Shewanella* regulates biofilm development [45]. In *Edwardsiella tarda*, 45 Kac proteins and 56 Ksu proteins have been linked to antimicrobial resistance (AMR) [46].

Lysine crotonylation (Kcr), a relatively new PTM, has been recognized for its evolutionary conservation and functional significance since its discovery in 2011 [14]. While many studies have elucidated its role in biological processes and human diseases [15,16], much of this research has focused on eukaryotic Kcr ''writers'' (crotonyltransferases) and ''erasers'' (decrotonylases) [20,22,23]. Recent advances in mass spectrometry have enabled the identification of thousands of Kcr sites in bacteria, yet their regulatory enzymes remain largely unexplored. The sole bacterial crotonyltransferase identified to date, Kct1, was discovered in *Streptomyces* through homology with eukaryotic GNATs. Conversely, the GNAT-domain-containing BspF in *Brucella* exhibits decrotonyltransferase activity, further complicating the classification of bacterial Kcr regulators [47]. The regulatory functions of Kcr in *S.pn* virulence and its “writers” and “erasers” have remained elusive.

Building on recent works identifying novel bacterial PTM enzymes [37,38,48], we successfully screened 13 GNAT-family proteins in *S.pn* D39 and identified SPD_0839 as a crotonyltransferase. Functional analysis confirmed SPD_0839′s enzymatic activity, catalyzing Kcr in vitro and in vivo. Our structural analysis revealed that SPD_0839′s core region harbors four conserved motifs (A–D), with Gly91 and Leu92 forming a β-bulge to stabilize tetrahedral reaction intermediates. Motif A, the most conserved, contains Phe93, Met94, and Val95, which participate in forming a V-shaped hydrophobic pocket that accommodates CrCoA. Phe93, critical for CrCoA binding via hydrogen bonds, is functionally analogous to Phe425 in Nmt1p, which uniquely contributes to acyl-CoA binding [49]. The mutation of Phe93 disrupts the binding pocket, highlighting the importance of specific hydrogen-bonding residues in GNAT function.

Many studies have reported that the activity of key enzymes in metabolic pathway are regulated through PTMs in bacteria, such as Kac and Khib negatively regulate the activity of ENO in *E. coli* [48], Kla reduced the activity of PykF [38]. Consistently, our findings demonstrate that SPD_0839-mediated Kcr regulates key enzymes in central carbon metabolism, including Pgi, Zwf, Pgk, Eno, Pyk, and PfkA. By reducing their activity, SPD_0839 negatively controls ATP production in *S.pn*. These results suggest that bacterial PTMs rapidly utilize available acyl-CoAs, such as CrCoA and AcCoA, to regulate metabolic pathways, maintaining fine-tuned control over bacterial growth and metabolism.

PLY, a major virulence factor in almost all *S.pn* serotypes, is a cholesterol-dependent cytolysin (CDC) that damages alveolar, endothelial, and cardiac cells by forming transmembrane pores. This activity disrupts host defenses, complement systems, and immune responses, facilitating bacterial dissemination. Intriguingly, sub-lytic concentrations of PLY activate host immune pathways, inducing inflammatory and cell repair responses. PLY's ability to form pores via oligomerization is crucial to its dual role in bacterial pathogenesis. Our study identifies K171 and K442 as key crotonylation sites on PLY, catalyzed by SPD_0839. Crotonylation enhances PLY's pore-forming activity and hemolytic capability, as demonstrated by the reduced activity of K171R and K442R mutants in vitro and in vivo. Biochemical and structural analyses revealed that these mutations destabilize PLY, reduce oligomer formation, and alter the interactions between domains 3 and 4. K171 is essential for transmembrane β-hairpin (TMH) formation, while K442 facilitates initial membrane binding. Decrotonylation disrupts these structural features, impairing PLY’s hemolytic activity. Notably, clinical multidrug-resistant *S.pn* strains exhibit low SPD_0839 expression (Supplementary Fig. 1g). This is consistent with observations of non-hemolytic *S.pn* variants, which promotes intracellular survival by minimizing inflammatory responses and tissue damage [50]. We speculate that decreased SPD_0839 expression in clinical *S.pn* strains represents an adaptive mechanism to enhance survival during infection.

In this study, we identified SPD_0839 as a novel crotonyltransferase in *S.pn*. SPD_0839 catalyzes Kcr at 205 sites on153 substrate proteins, including several key virulence factors such as PLY, PspA, CbpA, and SrtA. Structural analysis revealed that Phe93 plays a critical role in CrCoA binding and subsequent catalysis of SPD_0839. SPD_0839-mediated Kcr regulates ATP production by modifying key enzymes in central carbon metabolism. Importantly, it enhances PLY stability, oligomerization, and hemolytic activity through Kcr at K171 and K442. These findings provide valuable insights into the biological significance of Kcr in bacterial metabolism and virulence. SPD_0839 represents a promising target for developing therapeutic strategies against *S.pn* infection.

## Declaration of competing interest

The authors declare that they have no known competing financial interests or personal relationships that could have appeared to influence the work reported in this paper.
