# Supplementary material for: Crotonylation driving Streptococcus pneumoniae adaption and virulence
Source: J Adv Res. 2025 Jun 19;81:453–68. doi: 10.1016/j.jare.2025.06.045 (PMC12957837; doi:10.1016/j.jare.2025.06.045)
Supplement: Supplementary Data 1 [file mmc1.docx]

**Crotonylation driving *Streptococcu*s *pneumoniae* adaption and virulence**

Nan Li, Jianpeng Zhuang, Jiayi Wu, Zhuoti Xue, Jiayi Xu, Zuye Fang, Yundan Zheng, Yun Liu, Yunpeng Yang, Xinyu Ye, Qing-Yu He, Xuesong Sun

**Supplementary Information**

**This Supplementary Information file contains:**

Supplementary Figure 1. The Effect of Kcr on bacterial growth and preliminary exploration of SPD_0839 as a lysine crotonyltransferase.

Supplementary Figure 2. Conservation analysis of binding sites in SPD_0839.

Supplementary Figure 3. The effect of Phe93 on protein structure and function.

Supplementary Figure 4. MS/MS spectra of Kcr peptides identified in PLY.

Supplementary Figure 5. Purification and structural determination of PLY mutants.

Supplementary Figure 5. The effect of Kcr on the conformations of PLY monomers and oligomers.

Supplementary Figure 6. The effect of Kcr on the conformations of PLY monomers and oligomers.

Supplementary Table 1. 16S rRNA sequence similarity.

Supplementary Table 2. Strains and plasmids used in this study.

Supplementary Table 3. Primers used in this study.

**
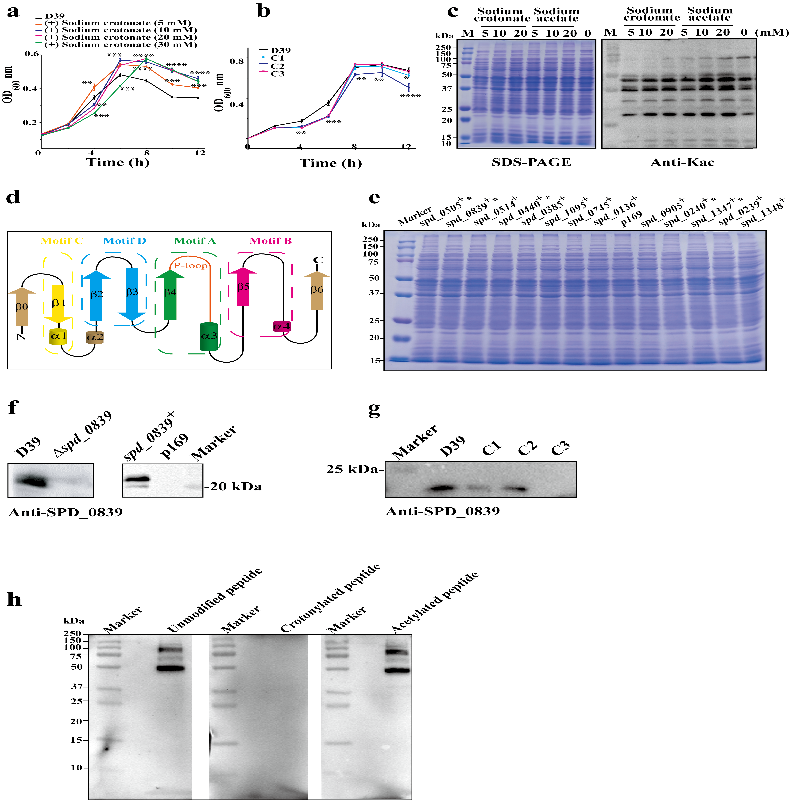
**

**Supplementary Figure 1. The Effect of Kcr on bacterial growth and preliminary exploration of SPD_0839 as a lysine crotonyltransferase. a.** Growth curves of *S.pn* D39 cultured in C+Y medium with varying concentrations of sodium crotonate. **b.** Growth curves of *S.pn* D39 compared with three clinical isolates. **c.** Western blot analysis of whole-cell lysates from D39 under different culture conditions using a pan-acetylation antibody. **d.** Schematic topology of GNAT domain showing conserved sequence motifs: C (β1–α1), D (β2–β3), A (β4–α3), and B (β5–α4). **e.** SDS-PAGE showing the loading control for whole-cell lysates from D39 strains containing overexpression plasmids. **f,** Western blot analysis using anti-SPD_0839 antibody to confirm SPD_0839 expression in Δ*spd_0839* and *spd_0839^+^*. **g.** Western blot analysis using anti-SPD_0839 antibody to confirm SPD_0839 expression in *S.pn* D39 compared with three clinical isolates. **h.** Anti-Kcr antibodies were treated with cotonylated peptide, acetylated or unmodified peptide to detect their specificity. In Figure a, b, n = 3, and Data are mean ± standard deviation (s. d). Unpaired student’s two-tailed t-test was applied to compare two experimental groups. Statistical significance was defined as *p<0.05, **p<0.01, ***p<0.001, ****p<0.0001, NS, no significance.


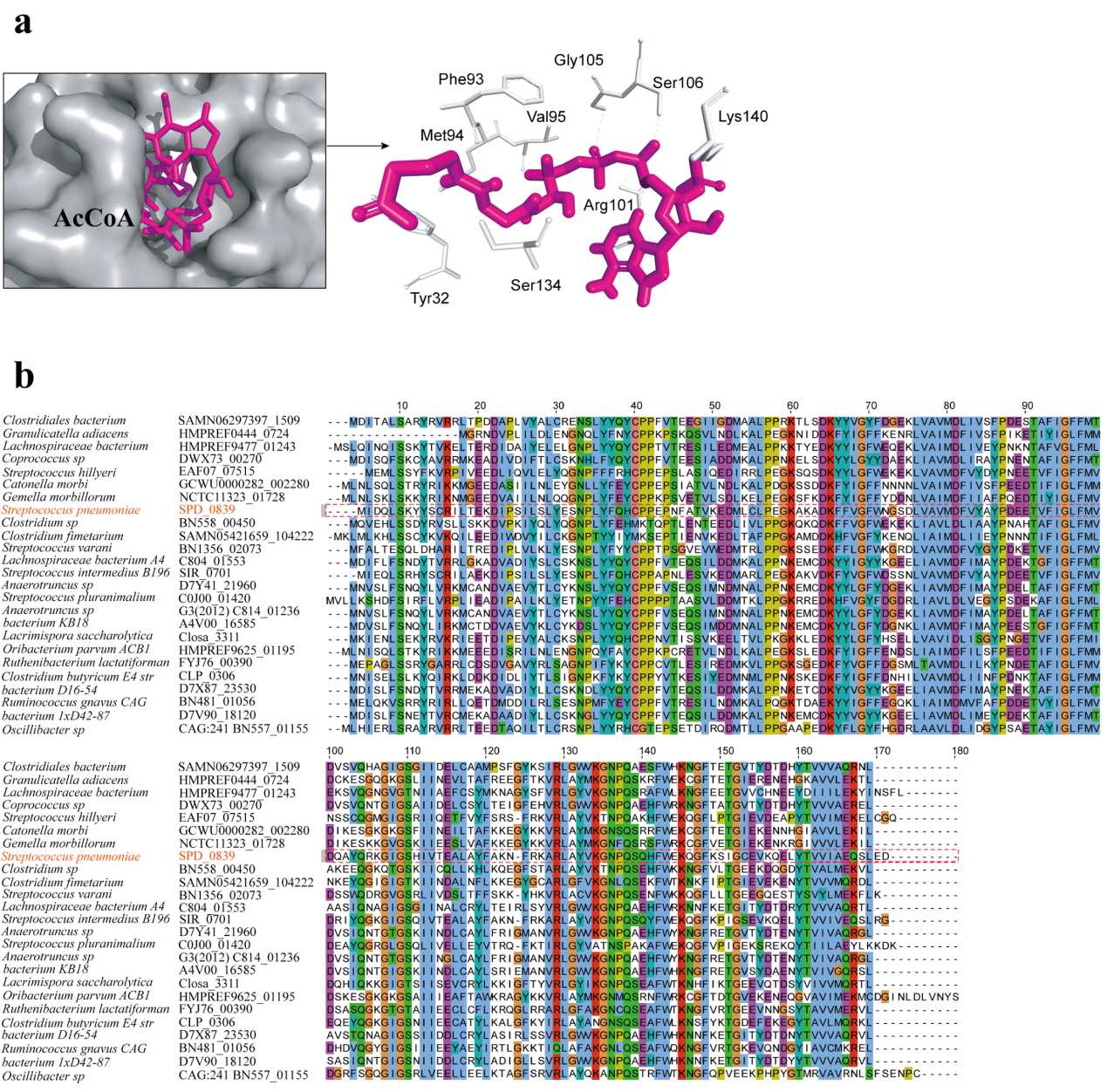


**Supplementary Figure 2.** **Conservation analysis of binding sites in SPD_0839. a.** Molecular docking showing AcCoA (purple stick) binding to SPD_0839. Amino acid residues involved in binding and the hydrophobic pocket formation are indicated. **b.** Multiple sequence alignment comparing SPD_0839 with homologous GNAT proteins across bacterial species, highlighting conserved regions.


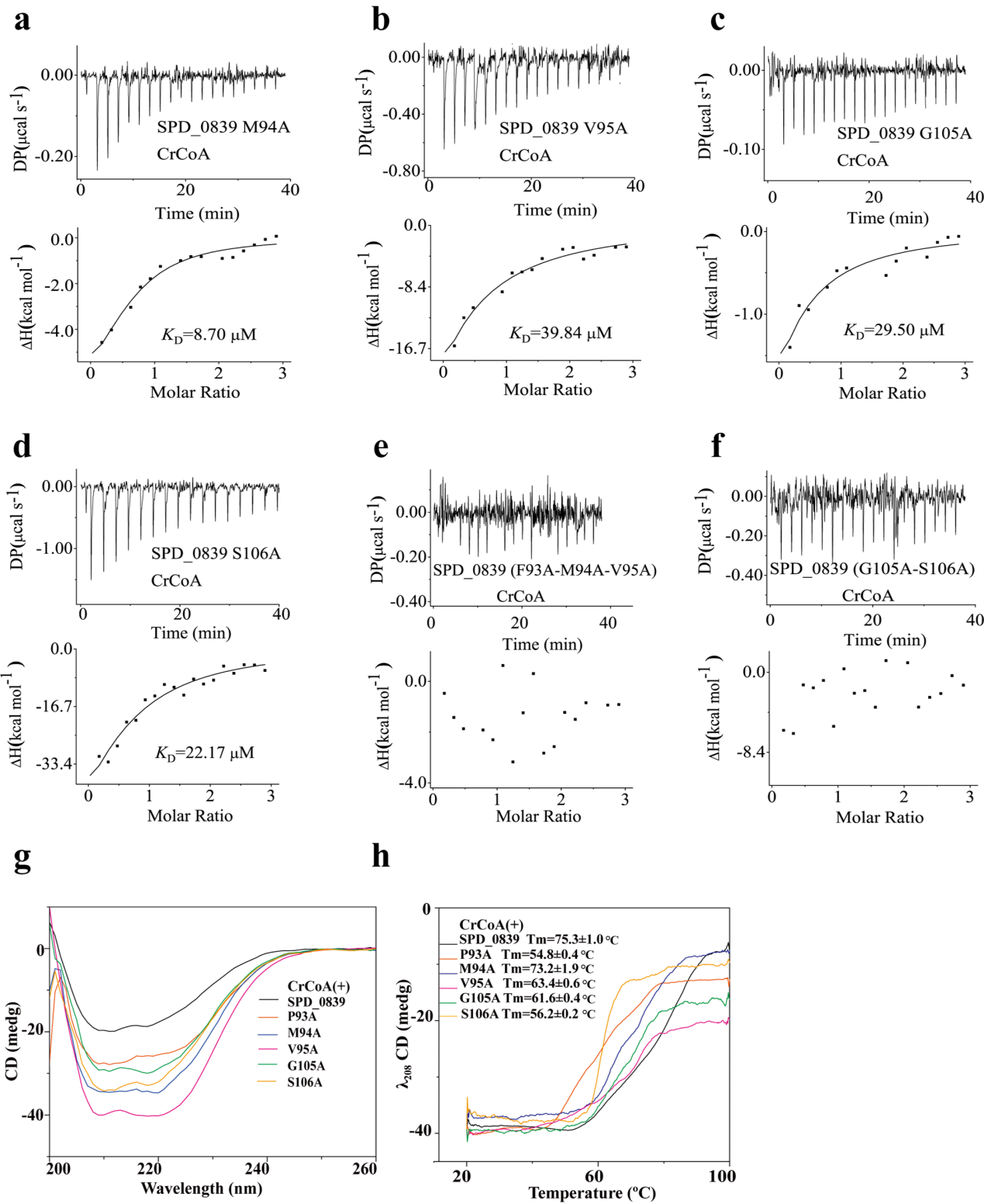


**Supplementary Figure 3. The effect of Phe93 on protein structure and function. a-f.** Isothermal titration calorimetry (ITC) analysis measuring the binding affinity of recombinant SPD_0839 mutant proteins for CrCoA. **g, h.** Circular dichroism (CD) analysis evaluating the conformation and thermal stability of SPD_0839 mutant proteins in complex with CrCoA.


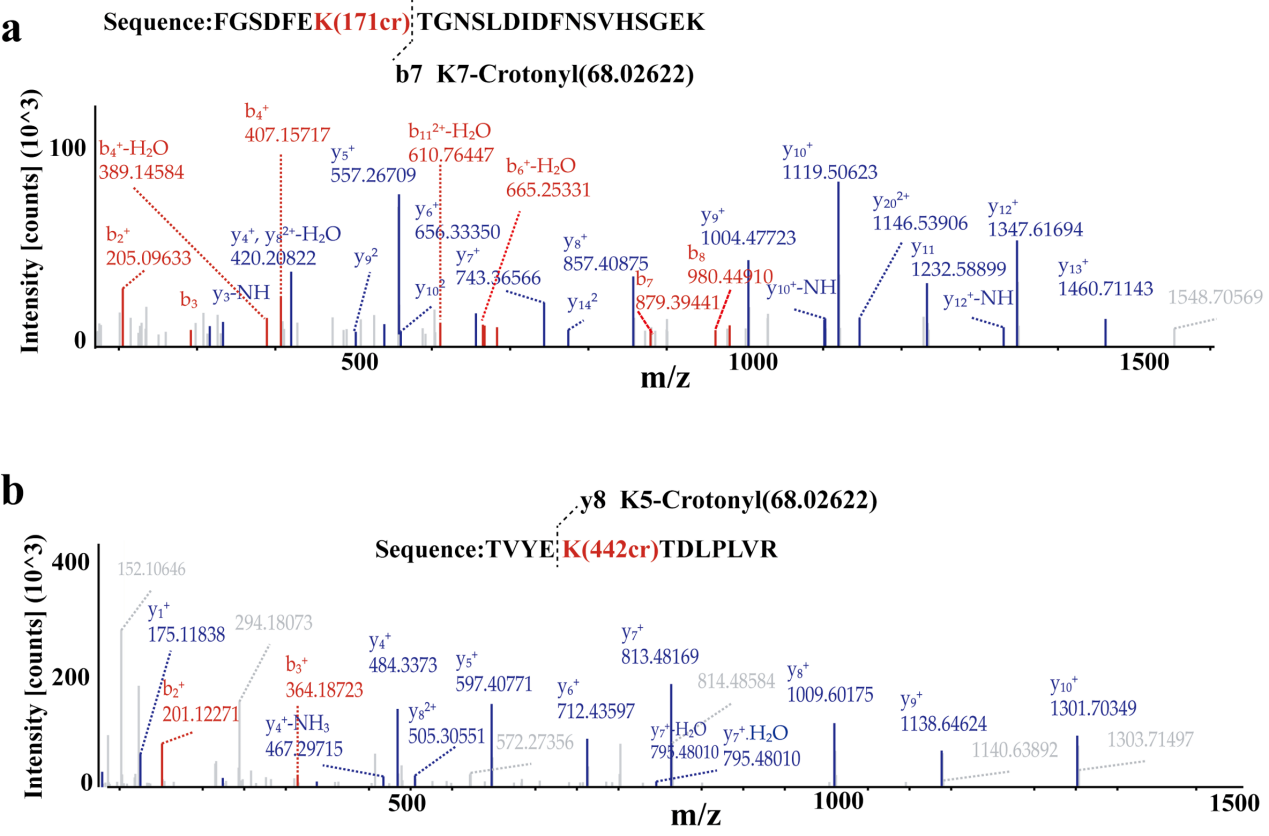


**Supplementary Figure 4. MS/MS spectra of Kcr peptides identified in PLY. a.** MS/MS spectrum of the peptide (FGSDFEK (171cr) TGNSLDIDFNSVHSGEK) from the *spd_0839^+^* proteome. **b.** MS/MS spectrum of the peptide (TVYEK (442cr) TDLPLVR) from the *spd_0839^+^* proteome.


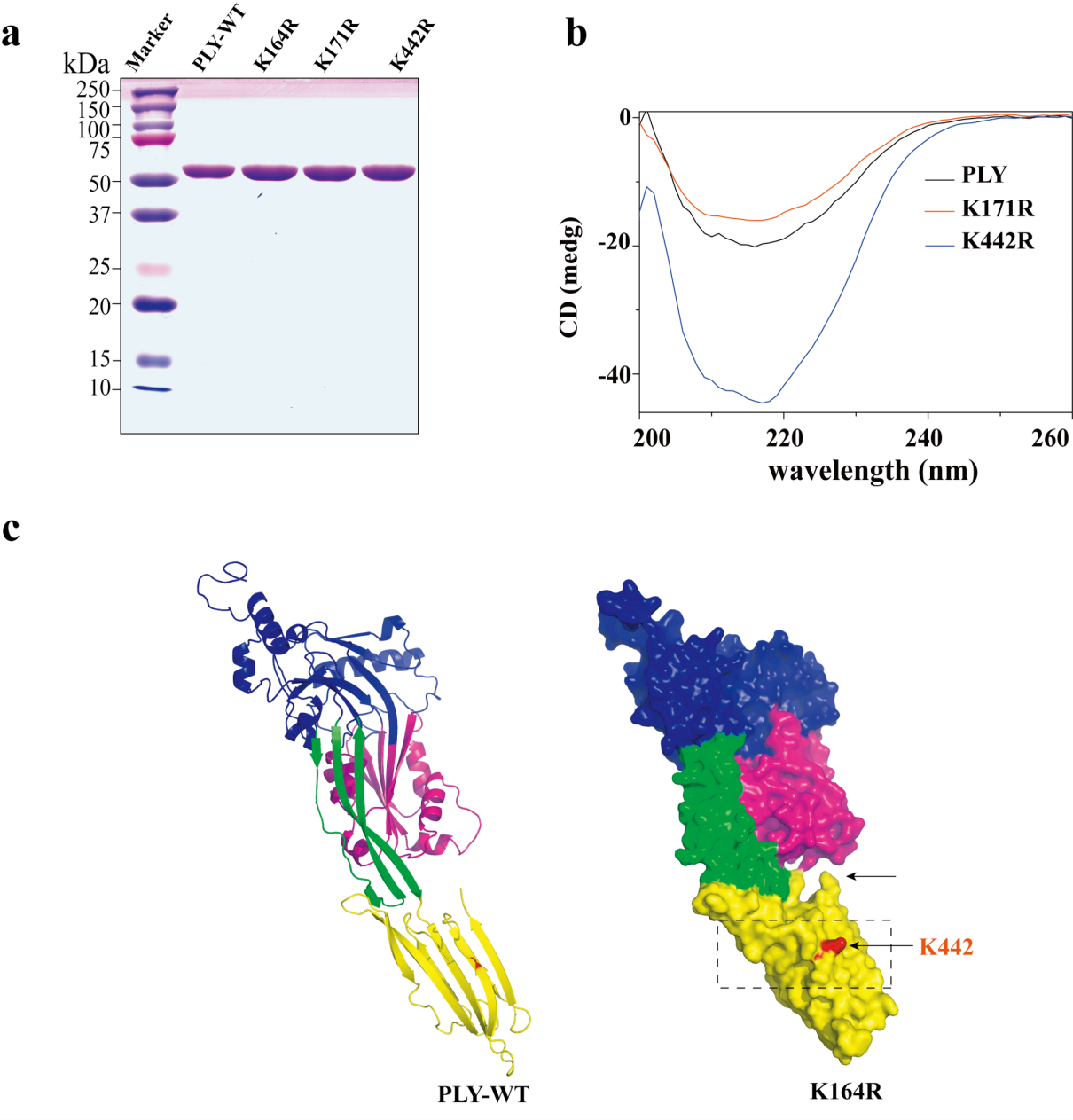


**Supplementary Figure 5. Purification and structural determination of PLY mutants. a.** SDS-PAGE analysis of purified PLY and its mutants. **b.** CD analysis to determine the secondary structure of WT PLY and its mutants. **c.** Cartoon and electron density maps of WT PLY and K164R mutants. Domains 1-4 are shown in different colors, with K442 in domain 4 highlighted in red. Arrows point to conformational changes at the junction between domains 3 and 4.


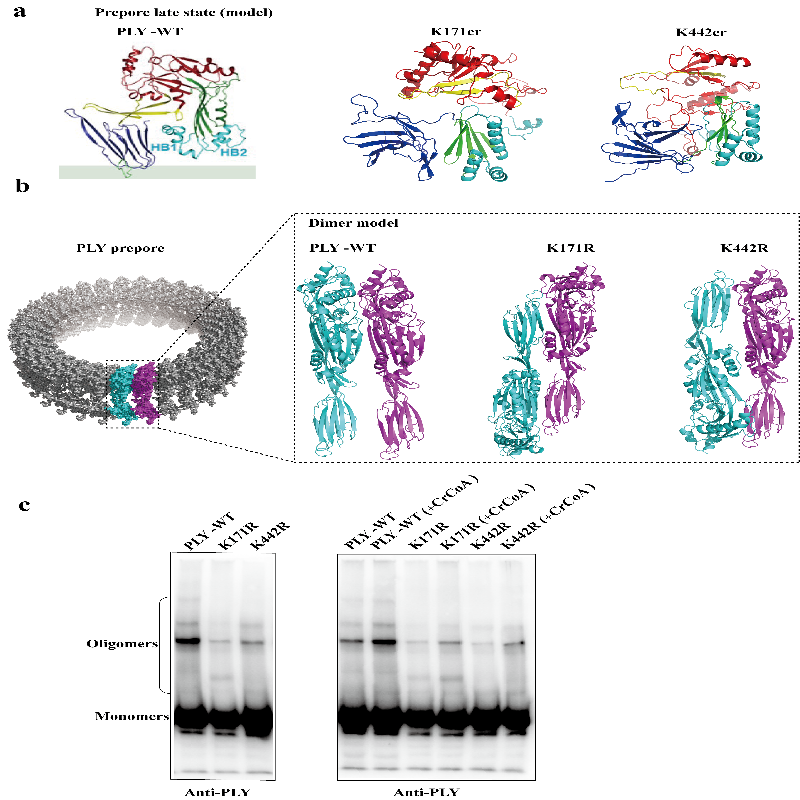


**Supplementary Figure 6. The effect of Kcr on the conformations of PLY monomers and oligomers.** **a**. Structural comparison between AlphaFold 3-predicted decrotonylated PLY dimer (K171/K442) and the experimentally observed PLY dimer conformation in the circular prepore assembly process. **b**. Western blot evaluation of PLY monomeric and oligomeric states in response to crotonylation/decrotonylation modifications.

**Supplementary Table 1.** **16S rRNA sequence similarity.**

| ***S.pn* strains** | **Clinical multidrug-resistant *S.pn* strains** | **Consistency of 16S rRNA sequence alignment** |
| --- | --- | --- |
| D39 | C1 | 97.51% |
|  | C2 | 97.84% |
|  | C3 | 94.55% |

**Supplementary Table 2**. **All bacterial strains and plasmids used in the study.**

| **Strains/plasmids** | **Description** | **Source/reference** |
| --- | --- | --- |

D39 Wild type ATCC (USA)

| Δ*spd_0839* | *spd_0839* mutant through homologous recombination derived from D39; Erm^r^ | This paper |
| --- | --- | --- |
| Δ*spd_0440* | *spd_0440* mutant through homologous recombination derived from D39; Erm^r^ | This paper |
| Δ*spd_0240* | *spd_0240* mutant through homologous recombination derived from D39; Erm^r^ | This paper |
| *p169*_*spd_0839* | D39 transformed with pIB-169_0839; Cm^r^ | This paper |
| *p169*_*spd_0505* | D39 transformed with pIB-169_0505; Cm^r^ | This paper |
| *p169*_*spd_0440* | D39 transformed with pIB-169_0440; Cmr | This paper |
| *p169*_*spd_0240* | D39 transformed with pIB-169_0240; Cm^r^ | This paper |
| *p169*_*spd_1347* | D39 transformed with pIB-169_1347; Cm^r^ | This paper |
| *p169*_*spd_1348* | D39 transformed with pIB-169_1348; Cm^r^ | This paper |
| *p169*_*spd_1095* | D39 transformed with pIB-169_1095; Cmr | This paper |
| *p169*_*spd_0905* | D39 transformed with pIB-169_0905; Cm^r^ | This paper |
| *p169*_*spd_1249* | D39 transformed with pIB-169_1249; Cmr | This paper |
| *p169*_*spd_0514* | D39 transformed with pIB-169_0514; Cm^r^ | This paper |
| *p169*_*spd_0909* | D39 transformed with pIB-169_0909; Cmr | This paper |
| *p169*_*spd_0239* | D39 transformed with pIB-169_0239; Cm^r^ | This paper |
| *p169*_*spd_0135* | D39 transformed with pIB-169_0135; Cm^r^ | This paper |
| *p169*_PLY | D39 transformed with pIB-169_PLY; Cm^r^ | This paper |
| *p169*_PLY^K164R^ | D39 transformed with pIB-169_PLY^K164R^; Cm^r^ | This paper |
| *p169*_PLY^K171R^ | D39 transformed with pIB-169_PLY^K171R^; Cm^r^ | This paper |
| *p169*_PLY^K442R^ | D39 transformed with pIB-169_PLY^K442R^; Cm^r^ | This paper |
| BL21 (lDE3) competent cells | Solarbio | Cat# C1400 |
| DH5α competent cells | Solarbio | Cat# C1100 |
| pIB169 vector | Shuttle plasmid contained Pveg promoter; Cm^r^ | ref^1^ |
| PGEX-4T-1 vector | Solarbio | Cat# VT000001 |
| pET28a vector | Solarbio | Cat# P3110 |
| PGEX-4T-1-SPD_0839 | *spd_0839* cloned into PGEX-4T-1; Amp^r^ | This paper |
| pET28a-SPD_0839 | *spd_0839* cloned into pET28a; Kan^r^ | This paper |
| pET28a-SPD_0839^P93A^ | *spd_0839* (93P→A) cloned into pET28a; Kan^r^ | This paper |
| pET28a-SPD_0839^M94A^ | *spd_0839* (94M→A) cloned into pET28a; Kan^r^ | This paper |
| pET28a-SPD_0839^V95A^ | *spd_0839* (95V→A) cloned into pET28a; Kan^r^ | This paper |
| pET28a-SPD_0839^G105A^ | *spd_0839* (105G→A) cloned into pET28a; Kan^r^ | This paper |
| pET28a-SPD_0839^S106A^ | *spd_0839* (106S→A) cloned into pET28a; Kan^r^ | This paper |
| pET28a-SPD_0839^P93A-M94A-V95A^ | *spd_0839* (93P-94M-95V→93A-94A-95A) cloned into pET28a; Kan^r^ | This paper |
| pET28a-SPD_0839^G105A-S106A^ | *spd_0839* (105G-106S→105A-106A) cloned into pET28a; Kan^r^ | This paper |
| pET28a-PLY | *Ply* cloned into pET28a; Kan^r^ | This paper |
| pET28a-PLY^K164R^ | *Ply*(164K→R) cloned into pET28a; Kan^r^ | This paper |
| pET28a-PLY^K171R^ | *Ply (*171K→R) cloned into pET28a; Kan^r^ | This paper |
| pET28a-PLY^K442R^ | *Ply* (424K→R) cloned into pET28a; Kan^r^ | This paper |

**Supplementary Table 3. All primers used in the study.**

| **Primer name Sequence (5’-3’)** | |
| --- | --- |
| *pET 28a-*SPD_0839-F  *pET28a-* SPD_0839-R  F93A-F  F93A-R  M94A-F  M94A-R  V95A-F  V95A-R  G105A-F  G105A-R  S106A-F  S106A-R  F93A/M94A/V95A-F  F93A/M94A/V95A-R G105A/S106A-F  G105A/S106A-R  *pGEX-4T-*SPD_0839-F  *pGEX-4T-*SPD_0839-R  *pET28a-*PLY-F  *pET28a-*PLY-R  K164R-F  K164R-R  K171R-F  K171R-R  K224R-F  K224R-R  K249R-F  K249R-R  K271R-F  K271R-R  K415R-F  K415R-R  K424R-F  K424R-R  K442R-F  K442R-R  p169_*spd_0839*-F  p169_*spd_0839*-R  p169_*spd_0505*-F  p169_*spd_0505*-R  p169_*spd_0440*-F  p169_*spd_0440*-R  p169_*spd_0240*-F  p169_*spd_0240*-R  p169_*spd_1347*-F  p169_*spd_1347*-R  p169_*spd_1348*-F  p169_*spd_1348*-R  p169_*spd_0239*-F  p169_*spd_0239*-R  p169_*spd_0514*-F  p169_*spd_0514*-R  p169_*spd_0905*-F  p169_*spd_0905*-R  p169_*spd_1095*-F  p169_*spd_1095*-R  p169_*spd_0135*-F  p169_*spd_0135*-R  p169_*spd_1249*-F  p169_*spd_1249*-R  p169_*spd_0909*-F  p169_*spd_0909*-R  Δ*spd_0839*-P1  Δ*spd_0839*-P2  Δ*spd_0839*-P3  Δ*spd_0839*-P4  Δ*spd_0505*-P1  Δ*spd_0505*-P2  Δ*spd_0505*-P3  Δ*spd_0505*-P4  Δ*spd_0440*-P1  Δ*spd_0440*-P2  Δ*spd_0440*-P3  Δ*spd_0440*-P4  Δ*spd_0240*-P1  Δ*spd_0240*-P2  Δ*spd_0240*-P3  Δ*spd_0240*-P4  Erm-F  Erm-R  pET28a-ENO-F  pET28a-ENO-R  pET28a-PfkA-F  pET28a-PfkA-R  pET28a-Zwf-F  pET28a-Zwf-R  pET28a-Pgi-F  pET28a-Pgi-R  pET28a-Pyk-F  pET28a-Pyk-R | **Primers for constructing SPD_0839 and its mutants expressing strains**  GGCGGATCCATGATTGACCAACTATCTAAG  CGCGAGCTCCTAATCTTCTAGGCTCTGTTC  GGCTATGGTTGATCAAGCCTATCAGAGGAAAGG  GCTTGATCAACCATAGCCAAACCAATAAAAACAGTCTCCTCA  GTTTGCGGTTGATCAAGCCTATCAGAGGA  GCTTGATCAACCGCAAACAAACCAATAAAAACAGTCTCCTC  TTATGGCTGATCAAGCCTATCAGAGGAAAGG  GGCTTGATCAGCCATAAACAAACCAATAAAAACAGTCT  GAAAGGGATTGCTAGTCATATTGTGACAGAAGCACTAGCT  GACTAGCAATCCCTTTCCTCTGATAGGCT  GAAAGGGATTGGTGCTCATATTGTGACAGAAGCACTAGCTTA  TGAGCACCAATCCCTTTCCTCTGATAGG  TTTGGCTGCGGCTGATCAAGCCTATCAGAGGAAAGG  TGATCAGCCGCAGCCAAACCAATAAAAACAGTCTCCTCA  GAAAGGGATTGCTGCTCATATTGTGACAGAAGCACTAGCTTA  TGAGCAGCAATCCCTTTCCTCTGATAGGCT  GCCGGATCCATGATTGACCAACTATCTAAG  GCCGTCGACCTAATCTT CTAGGCTCTGTTC  **Primers for constructing PLY and its Kcr sites mutants expressing strains**  CGCGGATCCATGGCAAATAAAGCAGTAAAT  CGCGAGCTCCTAGTCATTTTCTACCTTATCC  TCAAGGTCCGGTTTGGTTCTGACTTTGAAAAGACAG  AACCAAACCGGACCTTGAGTTGTTCCATGCTGT  ACTTTGAACGGACAGGGAATTCTCTTGATATTGATTT  TCCCTGTCCGTTCAAAGTCAGAACCAAACTTGACC  GGATTTACGACAGAGAGGAATTTCTGCAGAGCG  CCTCTCTGTCGTAAATCCTCTACCGTTACAGTATCTTGA  GTCTATCTCCGGTTGGAAACCACGAGTAAGAGTGA  TTCCAACCGGAGATAGACTTGGCGCCCATAAGC  TAAAAGGAGTCCGGGTAGCTCCTCAGACAGAGTGGA  CTACCCGGACTCCTTTTATCAAAGCTTCAAAAG  TTCCTTTACGAGGGAATGTTCGTAATCTCTCTGTC  CATTCCCTCGTAAAGGAATACTAGTGGTAAAGTGAGCC  CGTAATCTCTCTGTCCGAATTAGAGAGTGTACCGGGCTTGC  TCGGACAGAGAGATTACGAACATTCCCTTTTAA  CGGTTTATGAACGAACCGATTTGCCACTAGTGCG  CGGTTCGTTCATAAACCGTACGCCACCATTCCC  **Primers for constructing overexpression strains containing GNATs in *S.pn***  GGCGGATCCATGATTGACCAACTATCTAAG  CGCGTCGACCTAATCTTCTAGGCTCTGTTC  GGCGGATCCATGATTAGAAAAGTAGAAATGG  CGCGTCGAC TTACCTTAATTTCTTTCTCAG  GGCGGATCCTTGTGTTTTGTTCCTTATTA  CGCGTCGACTTAGACTAATTCCAATATAAAACT  GGCGGATCCATGATTACTATTAAAAAGCAA  CGCGTCGAC TTATTTTTTACGATTCATCCA  GCCGGATCCATGAAAATCAGACAAGCAAG  GCGGTCGAC TTATTTTTTCATTTCCCAGA  CCGGGATCCATGGAAATTCCAATTAAGAT  CGCGTCGACTCATTGTTCCTCCAGATAA  GGCGGATCCATGCCAGTAAATGAATATGG  GCGGTCGACTTAAAGTTCTCTCAAGCTCT  GCCGGATCCATGATTCAAGCAAGAAACAA  GCGGTCGACCTACACTTCGGAATCCCT  GGCGGATCCATGACAATTGAACTAAGAGATG  GGCGCGGTCGACTTATTTCTCAATTGTTAA  GCGCGCGGATCCATGATTTATTTAAGAAAG  GCGGTCGACTCAGGAGTTTCCATTGATTTG  GGCGGATCCATGATAGAAATCAAACGAATC  CGCGTCGACCTATCCTTCATCTATTTCTCT  GCCGGTACCATGAAATCTATCGGTACGC  CGCGTCGAC TCAACATTCTTCCCTACTTAT  GGCGGATCCATGCTAAGAGATTTGCAAG  CGCGTCGAC CTAAAAGATGCGAATAAACC  **Primers for constructing overexpression strains containing GNATs in *S.pn***  TTGGGCCAGCAGGTACAGGGAA  CCCGCACTTCAACCATCGCCCT  ATCAAACAAATTTTGGGCCCGGCTTTTCAAATTCCTACTTTCTT  ATTCTATGAGTCGCTGCCGACTAAATGGCATCAAGTAAGAACTA  TTCTGCTGAGATGCGTCCAATC  CAGAAAGGGCATCTGCCGCAGC  ATCAAACAAATTTTGGGCCCGGTATTTAAACTGTTCTGAGAAGC  ATTCTATGAGTCGCTGCCGACTGAAGAGGATTCTTCTATTGAAA  CGAGCCCTCACACGTCTGATTG  CCTCGCTGGTTTCAGCGTCGTC  ATCAAACAAATTTTGGGCCCGGTGCACTACCTTTTCAACAAAAG  ATTCTATGAGTCGCTGCCGACTTCCTGCCACTTTCTCCCCCTTA  TTGCAAGAAATTGCTCTAGCGG  CAACCGCTGTACGACAAGACGG  ATCAAACAAATTTTGGGCCCGGCTCAACACCTCTTAAAGTTCTC  ATTCTATGAGTCGCTGCCGACTCAAAACTTGTTTTTTCTTAAGC  CCGGGCCCAAAATTTGTTTGAT  AGTCGGCAGCGACTCATAGAAT  **Primers for constructing endogenous substrate protein expressing strains**  CGCGGATCCATGTCAATTATTACTGATG  CGCGAGCTCTTATTTTTTAAGGTTGTAGAATG  CGCGGATCCATGAAACGTATTGCTG  CGGGAGCTCTTATGACAAGCTCTTATTCAAGC  GCCGGATCCATGTCATCTAAGGTTATTGTTACA  CGCGTCGAC TTATTCTAAGCGACCATCTTGA  CGCGCCGGATCCATGTCACATATTAAATTTGA  CGCGTCGACTTATAGACGTGCGTTAAGTTC  CGCGCCGGATCCATGAACAAACGTGT  CGCGTCGACTTAACGTACTGTGCGGATACGC |

**Reference:**

1. Biswas, I., Jha, J.K. & Fromm, N. Shuttle expression plasmids for genetic studies in Streptococcus mutans. *Microbiology* **154**, 2275-2282 (2008).
